# AI‐Designed TREM1‐Targeted LYTAC Nanoparticles Reprogram the Neuroimmune Microenvironment in Traumatic Brain Injury

**DOI:** 10.1002/advs.77972

**Published:** 2026-09-27

**Authors:** Yimin Huang, Yuan Liu, Huan Peng, Yu Ni, Zhengqiao Jiang, Chenxuan Yu, Xincheng Zhang, Huayu Kang, Yanchao Liu, Juan Chen, Kai Zhao, Lin Han, Xin Zou, Kai Shu, Xiaochuan Wang, Jian‐Zhi Wang, Ting Lei, Chao Gan, Jianfeng Liu, Huaqiu Zhang

**Affiliations:** ^1^ Department of Neurosurgery Tongji Hospital of Tongji Medical College of Huazhong University of Science and Technology Wuhan Hubei People's Republic of China; ^2^ Hubei Key Laboratory of Neural Injury and Functional Reconstruction Huazhong University of Science and Technology Wuhan People's Republic of China; ^3^ College of Life Science and Technology Huazhong University of Science and Technology Wuhan People's Republic of China; ^4^ Institute of Integrated Traditional Chinese and Western Medicine Tongji Hospital of Tongji Medical College of Huazhong University of Science and Technology Wuhan People's Republic of China; ^5^ Department of Pathophysiology School of Basic Medicine Key Laboratory of Ministry of Education of China for Neurological Disorders, Tongji Medical College Huazhong University of Science and Technology Wuhan Hubei People's Republic of China

**Keywords:** LYTAC, macrophage/monocyte, nanoparticles, traumatic brain injury, TREM1

## Abstract

Secondary neuroinflammation drives progressive damage after traumatic brain injury (TBI), but therapeutics that modulate key inflammatory amplifiers remain limited. Here, we identify infiltrating macrophages as the predominant TREM1‐expressing population in TBI human and mouse brain and show that TREM1 deletion attenuates neuroinflammation, preserves neurovascular integrity, and improves neurological and behavioral recovery after TBI. To develop a pharmacological strategy, we designed a de novo TREM1‐binding peptide, converted it into a mannose‐6‐phosphate lysosome‐targeting chimera (LYTAC), and delivered it using an Angiopep‐2‐functionalized, pH‐responsive dendrimer nanoparticle. This Angiopep‐2/PAMAM/TPA/LYTAC nanoparticle (APTL‐NP) enhanced brain delivery, promoted lesion‐responsive payload release, reduced TREM1 protein abundance in vivo, and outperformed free degrader and LP17 in suppressing inflammatory mediators, edema, blood–brain barrier disruption, neuronal apoptosis, and behavioral deficits. Single‐cell transcriptomics suggested remodeling of myeloid, astrocytic, vascular, and SPP1/CXCL‐associated communication programs. These findings support targeted TREM1 reduction as a strategy for modulating secondary TBI injury.

## Graphical abstract

AI‐enabled de novo design of a TREM1 binder, integrated into a BBB‐crossing and macrophage‐targeted nanoplatform, enables TREM1 degradation and attenuates neuroinflammation, thereby promoting neuroprotection and tissue repair after traumatic brain injury.

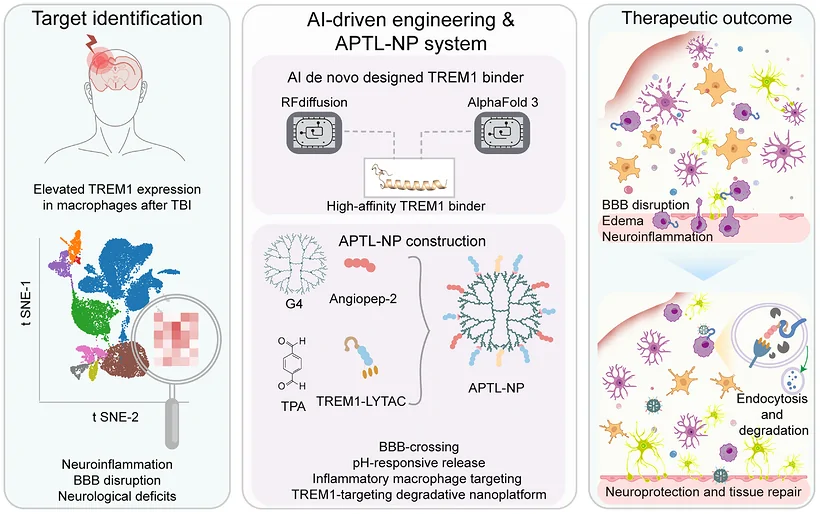

## 1 Introduction

Traumatic brain injury (TBI) is a major cause of death and long‐term neurological disability worldwide. Its pathological evolution involves an immediate primary mechanical insult followed by a delayed and potentially modifiable secondary injury cascade, including neuroinflammation, blood–brain barrier (BBB) disruption, cerebral edema, neuronal apoptosis, and progressive neurological dysfunction [1, 2, 3]. Among these processes, maladaptive neuroinflammation is increasingly recognized as a central driver of secondary injury. Although early inflammatory responses contribute to debris clearance and tissue repair, excessive or unresolved activation of innate immune pathways promotes cytokine and chemokine production, peripheral immune‐cell infiltration, vascular leakage, and neuronal damage, thereby sustaining a detrimental post‐traumatic microenvironment [4, 5]. Despite advances in neurocritical care, effective disease‐modifying pharmacotherapies that interrupt this inflammatory cascade remain unavailable. This unmet need reflects both an incomplete understanding of the dominant molecular amplifiers that sustain post‐traumatic inflammation and the difficulty of delivering biologically active therapeutics to disease‐relevant cells within the injured central nervous system [6, 7].

Triggering receptor expressed on myeloid cells‐1 (TREM1) is an innate immune receptor expressed mainly by neutrophils, monocytes, and macrophages and functions as a potent amplifier of inflammatory signaling [8, 9, 10]. Through its adaptor protein DAP12, TREM1 cooperates with Toll‐like receptors and other pattern‐recognition pathways to enhance downstream inflammatory activation, thereby reinforcing feed‐forward cytokine responses [11, 12, 13]. This signaling property makes TREM1 highly relevant to sterile inflammatory conditions such as TBI, in which damage‐associated molecular patterns released from injured neural and vascular tissues can sustain peripheral myeloid‐cell activation. However, the cellular origin of TREM1‐expressing cells in the injured brain and the functional contribution of TREM1 to post‐traumatic neurovascular damage have not been fully defined. In this study, analysis of human TBI samples, murine controlled cortical impact models, single‐cell transcriptomic datasets, Cx3cr1/Ccr2 reporter mice, and bone marrow chimeras identified infiltrating peripheral macrophages as the predominant TREM1‐expressing population in the TBI brain. Genetic ablation of Trem1 attenuated inflammatory activation, preserved BBB and neuronal integrity, reduced edema and apoptosis, and improved neurological and behavioral recovery after TBI. These findings support TREM1‐positive infiltrating myeloid cells as a therapeutically actionable driver of secondary brain injury.

Translating the protective phenotype of Trem1 deficiency into a pharmacological strategy remains challenging. Existing TREM1‐targeting approaches, including the LP17 decoy peptide, rely primarily on occupancy‐based inhibition and therefore require sustained exposure to maintain receptor or ligand blockade [14, 15, 16, 17]. Such a requirement is difficult to satisfy in TBI because peptide therapeutics often exhibit rapid systemic clearance, limited brain accumulation, and insufficient retention within the lesion microenvironment. In addition, transient receptor blockade does not necessarily reproduce the loss‐of‐function state achieved by genetic deletion and may permit signaling recovery once drug concentrations decline [18]. These limitations suggest that a strategy capable of reducing TREM1 protein abundance, rather than only blocking receptor activation, may provide a more durable means of suppressing TREM1‐dependent inflammatory amplification.

The convergence of generative artificial intelligence (AI) and targeted protein degradation (TPD) offers a powerful avenue to address this challenge [19, 20, 21]. Lysosome‐targeting chimeras (LYTACs) utilize bifunctional molecules to bridge a cell‐surface protein of interest to a lysosomal trafficking receptor, triggering its internalization and degradation [22, 23]. However, the development of LYTACs for TBI faces two hurdles: the need for ultra‐high‐affinity binders that exceed the performance of natural ligands, and the requirement for a delivery vehicle capable of crossing the BBB and releasing its payload specifically within the inflamed microenvironment [24, 25, 26]. Recent breakthroughs in deep learning, specifically protein “hallucination” algorithms like RFdiffusion, now enable the *de novo* design of binders with optimized interaction geometries, while advances in stimuli‐responsive nanomedicine allow for precise spatiotemporal drug release [27, 28].

In this study, we delineated the cellular origin of TBI‐associated TREM1 and identified infiltrating peripheral macrophages as the predominant TREM1‐expressing population in the TBI brain. Building on this finding, we used generative deep learning to design a de novo TREM1‐binding peptide and engineered it into a lysosome‐targeting chimera designed to reduce cell‐surface TREM1 abundance. To improve brain delivery and lesion‐responsive release, we incorporated this degrader into an Angiopep‐2‐functionalized, pH‐responsive dendrimer nanoplatform, termed Angiopep‐2/PAMAM/TPA/LYTAC nanoparticle (APTL‐NP). We show that APTL‐NP crosses the BBB, accumulates in the injured brain, targets inflammatory myeloid cells, and reduces TREM1 protein abundance in vivo. Functionally, APTL‐NP suppresses post‐traumatic neuroinflammation, preserves blood‐brain barrier and neuronal integrity, and improves neurological recovery after TBI. Single‐cell transcriptomic profiling further indicates that APTL‐NP treatment reshapes TBI‐associated myeloid, astrocytic, and vascular responses and attenuates inflammatory cell–cell communication programs, including SPP1‐ and CXCL‐associated signaling. Together, these findings establish a therapeutic framework that integrates AI‐guided binder design, targeted receptor degradation, and BBB‐oriented nanodelivery to modulate secondary neuroinflammation after traumatic brain injury.

## 2 Results

### 2.1 TREM1 Is Predominantly Upregulated in Infiltrating Myeloid Cells After TBI

**FIGURE 1:**
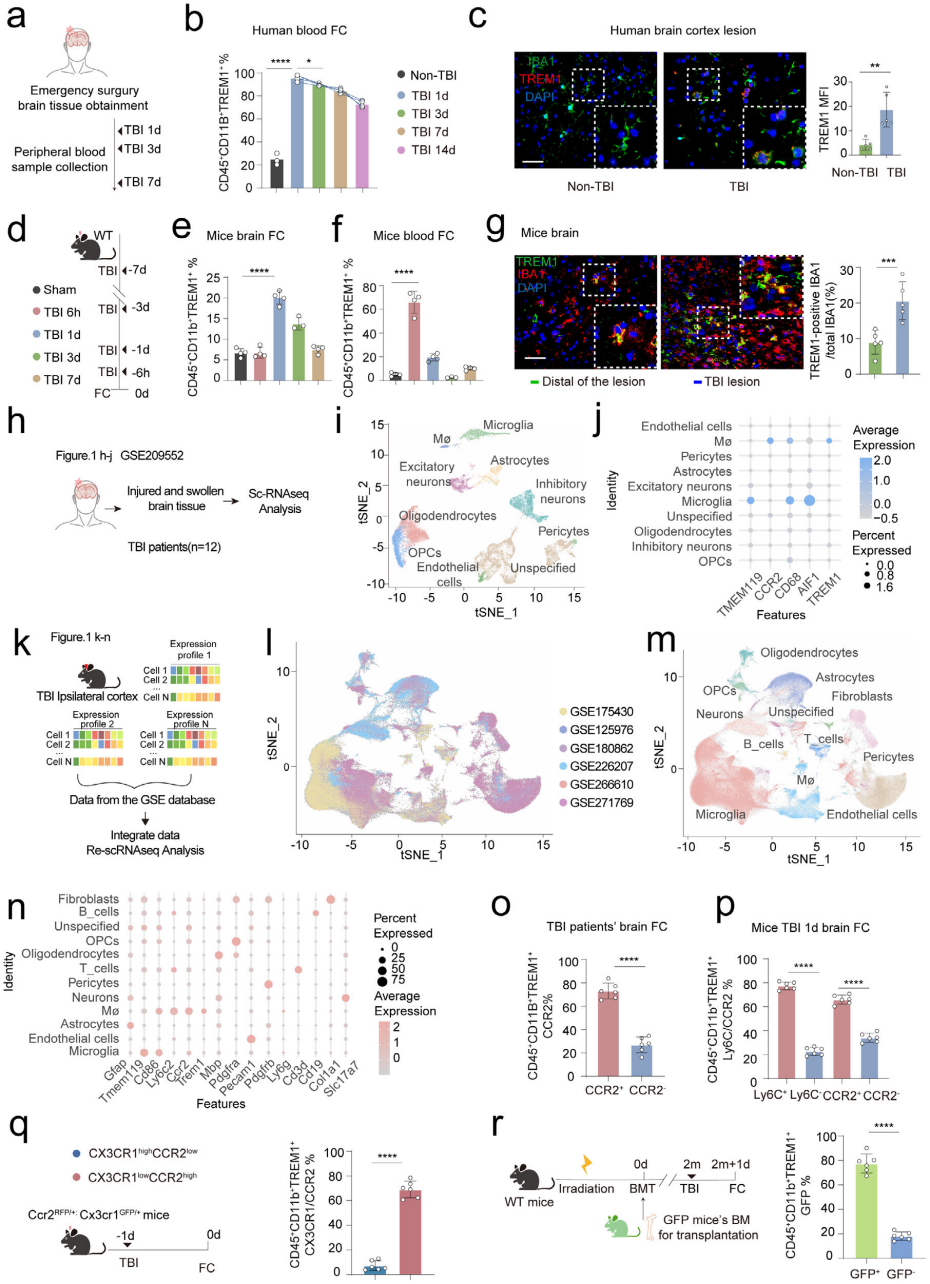
TREM1 is predominantly upregulated in infiltrating peripheral myeloid cells. (a–c) Spatiotemporal dynamics of TREM1 expression in clinical TBI samples. (a) Schematic of the clinical study design involving peripheral blood and brain tissue collection from TBI patients. (b) Flow cytometric quantification of circulating CD45^+^CD11b^+^TREM1^+^ myeloid cells, revealing an acute systemic surge at 1 day post‐TBI (*n* = 3 patients per time point, one‐way ANOVA followed by Tukey's multiple‐comparison test). (c) Representative immunofluorescence images and quantification of TREM1 expression in IBA1^+^ myeloid cells in lesional and non‐lesional surgical cortical tissues from TBI patients (scale bar, 100 µm, *n* = 3, two‐tailed paired Student's *t* test). (d–g) Temporal characterization of TREM1 expression in a murine TBI model. (d) Experimental timeline. (e, f) Flow‐cytometric quantification of CD45^+^CD11b^+^TREM1^+^ myeloid cells in mouse brain (e) and peripheral blood (f) (*n* = 4 mice per time point, one‐way ANOVA followed by Tukey's multiple‐comparison test). (g) Representative immunofluorescence images and quantification of TREM1 expression in the perilesional and distal cortical regions at 1 day post‐TBI (*n* = 5 mice; scale bar, 100 µm, two‐tailed paired Student's *t* test). (h–n) Single‐cell transcriptomic landscape of TREM1^+^ populations. (h–j) Reanalysis of human TBI tissue single‐cell RNA‐sequencing data (GSE209552). i, t‐SNE visualization of major cell types. (j) Dot plot identifying TREM1 and CCR2 enrichment specifically in the Macrophage (Mφ) and Microglia clusters. (k–n) Integrated meta‐analysis of murine TBI scRNA‐seq datasets. (l) Batch‐corrected t‐SNE map of integrated samples. m, Cell type annotation. n, Marker gene expression profiling reveals that Trem1 is predominantly expressed in infiltrating myeloid subsets (Macrophage) rather than homeostatic microglia. (o–r) Lineage tracing identifies infiltrating monocytes as the primary source of TREM1. (o) Flow cytometric analysis of human TBI brain tissue showing that TREM1^+^ cells predominantly co‐express the monocyte marker CCR2 (*n* = 6 patients, two‐tailed Student's *t* test). (p) Characterization of brain single‐cell suspensions at 1 day post‐TBI. The majority of TREM1^+^ cells are Ly6C^+^ and CCR2^+^, indicative of inflammatory monocytes (*n* = 6 mice per group, unpaired two‐tailed Student's *t* test). (q) Fate mapping using Ccr2^RFP/+^: Cx3cr1^GFP/+^ reporter mice. TREM1 is highly enriched in the infiltrating monocyte population CX3CR1^low^CCR2^high^ compared to resident microglia CX3CR1^high^CCR2^low^ (*n* = 6 mice per group, one‐way ANOVA followed by Tukey's multiple‐comparison test). (r) Bone marrow transplantation (BMT) chimera analysis. Head‐protected WT recipients reconstituted with GFP^+^ bone marrow showed that TREM1^+^ cells in the injured brain were predominantly GFP^+^ bone‐marrow‐derived infiltrating cells rather than GFP^−^ host‐resident cells (*n* = 6 mice per group, unpaired two‐tailed Student's t test). Data are presented as mean ± SD. Each dot represents an individual patient or mouse, as applicable. Statistical tests are specified for each panel above. Exact *p* values are provided in the Source Data. ^*^ *p* < 0.05, ^**^ *p* < 0.01, ^***^ *p* < 0.001, ^****^ *p* < 0.0001.

Peripheral blood from TBI patients exhibited significantly elevated levels of CD45^+^CD11b^+^TREM1^+^ myeloid cells on day 1 post‐TBI (Figure 1a, Figure S1a,b, and Table S1). Longitudinal analysis revealed these TREM1^+^ myeloid cells peaked at day 1 and subsequently declined over time (Figure 1b). Furthermore, in traumatic brain specimens from TBI patients, IBA1^+^ cells showed increased TREM1 expression compared to non‐lesional surgical control tissue (Figure 1c).

Subsequently, we assessed the temporal dynamics of TREM1 expression in the controlled cortical impact (CCI) mouse model after TBI (Figure 1d). Flow cytometric analysis revealed a marked increase in CD45^+^CD11b^+^TREM1^+^ cells within the injured brain at 1 day post‐TBI (Figure 1e and Figure S1c), whereas the corresponding population in peripheral blood increased as early as 6 h after TBI (Figure 1f and Figure S1d,e). Immunofluorescence staining further showed that TREM1^+^ cells were predominantly localized in the perilesional region of mouse TBI brains (Figure 1g). Consistent with the findings in human TBI specimens, TREM1^+^IBA1^+^ cells in mouse lesions displayed an amoeboid morphology, suggesting activation of myeloid‐lineage cells after TBI (Figure 1c,g). Because IBA1 is expressed by both resident microglia and infiltrating macrophages, we next asked whether the TREM1^+^IBA1^+^ population was preferentially associated with microglia or monocyte‐derived macrophages. In silico analysis of single‐cell RNA‐sequencing data from swollen brain tissue of patients with TBI (GSE209552) showed that TREM1 was predominantly enriched in the monocyte–macrophage compartment. TREM1 expression overlapped with the myeloid and macrophage‐associated markers CCR2, CD68, and AIF1, but showed little overlap with TMEM119, a marker enriched in resident microglia (Figure 1h–j). We further integrated and reanalyzed multiple publicly available single‐cell RNA‐seq datasets from mouse TBI models, focusing on cells isolated from the ipsilateral cerebral cortex after TBI. Integrated tSNE visualization resolved major cortical cell populations, including microglia, monocyte‐derived macrophages (Mø), astrocytes, oligodendrocytes, oligodendrocyte precursor cells, endothelial cells, pericytes, fibroblasts, and T and B lymphocytes (Figure 1k–m). Feature plot analysis further demonstrated that Trem1 was highly enriched in Mø expressing Cd86, Ccr2, and Ly6c2, whereas its expression was low in neurons, oligodendrocytes, and endothelial cells (Figure 1n). Collectively, these findings indicate that TBI‐associated TREM1 expression is predominantly linked to the myeloid/monocyte–macrophage lineage after TBI.

Next, we performed flow cytometry analysis of TBI patient brain lesions, over 70% of CD45^+^CD11b^+^TREM1^+^ cells were CCR2‐positive (Figure 1o). To further substantiate these findings, flow cytometry analysis of murine TBI brain samples showed that over 80% of CD11b^+^CD45^+^TREM1^+^ cells were Ly6C‐positive, and approximately 70% were CCR2‐positive, indicative of peripheral macrophage markers (Figure 1p). Similarly, using Cx3cr1^Gfp/+^: Ccr2^Rfp/+^ mice, approximately 70% of TREM1^+^ cells exhibited a CX3CR1^low^CCR2^high^ phenotype (Figure 1q). To determine whether TREM1^+^ cells were derived from the periphery, we generated head‐protected GFP bone marrow chimeras before TBI. Flow cytometric analysis showed that nearly 80% of TREM1^+^ cells in the injured brain were CD45^+^CD11b^+^GFP^+^ (Figure 1r).

These data together indicated that TREM1 elevation in the TBI brain was predominantly derived from peripheral macrophages.

### 2.2 Systemic TREM1 Ablation Attenuates TBI‐Induced Neuroinflammation, BBB Disruption, and Neurological Deficits

**FIGURE 2:**
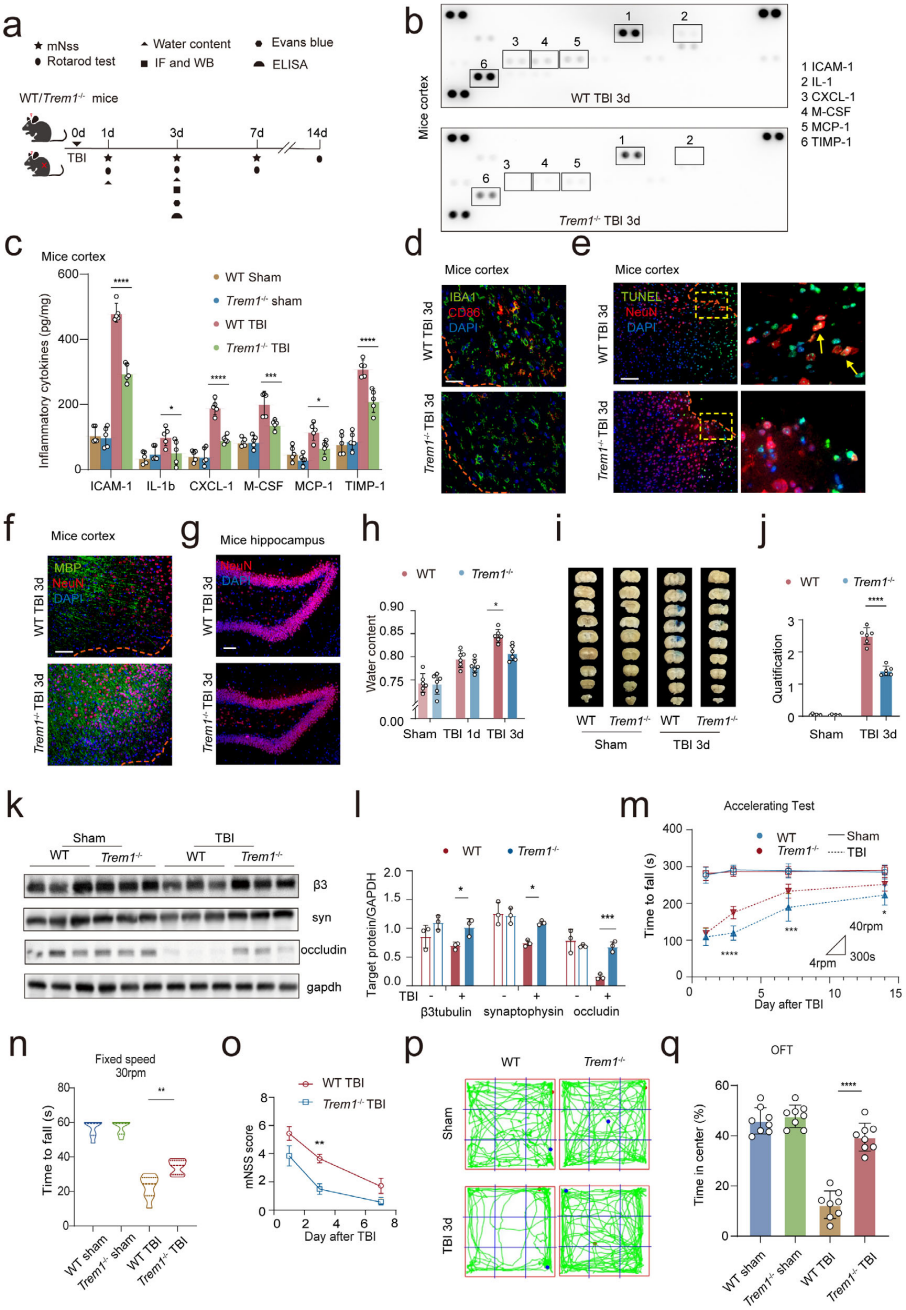
Systemic TREM1 ablation attenuates neuroinflammation and neurological deficits after TBI. (a) Schematic timeline of the experimental design. WT and *Trem1* ^−/−^ mice were subjected to CCI or sham surgery, followed by longitudinal behavioral and histological assessments. (b, c) Analysis of the neuroinflammatory profile in the perilesional cortex at 3 days post‐TBI. (b) Representative images of inflammatory cytokines in TBI lesions detected by the Proteome Profiler Array Mouse Cytokine Array Panel A in WT and *Trem1* ^−/−^ mice at 3 days post‐TBI. (c) Quantification of inflammatory cytokines in TBI lesions of WT and *Trem1* ^−/−^ mice at 3 days post‐TBI or sham treatment by ELISA (*n* = 5 mice per group, two‐way ANOVA followed by Tukey's multiple‐comparison test). (d) Representative immunofluorescence images of macrophages (IBA1^+^, green) and the reactivation marker CD86 (red) in the TBI cortex. Trem1 ablation significantly reduced reactive polarization (scale bar, 50 µm). (e) Evaluation of neuronal apoptosis. Representative co‐staining of TUNEL (green) and NeuN (red) (left) (*n* = 3 mice per group, scale bar, 100 µm). Yellow arrows indicate apoptotic neurons. (f) Assessment of white matter integrity. Representative images of Myelin Basic Protein (MBP, green) and NeuN (red) (left) and quantification of MBP fluorescence intensity (right) (scale bar, 100 µm). (g) Representative immunofluorescence images of hippocampal neurons (NeuN, red) showing preserved cellular density in *Trem1* ^−/−^ mice (*n* = 3 mice per group; scale bar, 100 µm). (h) Brain water content analysis at 1day and 3 d post‐TBI, indicating alleviated cerebral edema in *Trem1* ^−/−^ mice (*n* = 6 mice per group; two‐way ANOVA followed by Tukey's multiple‐comparison test). (i, j) Assessment of blood‐brain barrier (BBB) permeability. (i) Representative images of Evans Blue extravasation. (j) Quantification of dye leakage (*n* = 6 mice per group; two‐way ANOVA followed by Tukey's multiple‐comparison test). (k, l) Molecular validation of tissue preservation. (k) Representative Western blots of the neuronal marker beta3‐tubulin, synaptic marker Synaptophysin (Syn), and tight junction protein Occludin. l, Densitometric quantification normalized to GAPDH (*n* = 3 independent experimental replicates; two‐way ANOVA followed by Tukey's multiple‐comparison test). (m–o) Assessment of sensorimotor function. m, Latency to fall in the accelerating Rotarod test (*n* = 8 mice per group; two‐way ANOVA followed by Tukey's multiple‐comparison test). (n) Performance in the fixed‐speed (30 rpm) Rotarod test (*n* = 8 mice per group, one‐way ANOVA followed by Tukey's multiple‐comparison test). o, Modified Neurological Severity Score (mNSS) trajectory (*n* = 8 mice per group; two‐way ANOVA followed by Tukey's multiple‐comparison test). (p, q) Evaluation of anxiety‐like behavior and exploratory activity in the Open Field Test (OFT). (p) Representative locomotor tracking plots. (q) Quantification of the percentage of time spent in the center zone (*n* = 8 per group; two‐way ANOVA followed by Tukey's multiple‐comparison test). Data are presented as mean ± SD. Each dot represents an individual mouse, except in l, where each dot represents an independent experimental replicate. Statistical tests are specified for each panel above. Exact *p* values are provided in the Source Data. ^*^ *p* < 0.05, ^**^ *p* < 0.01, ^***^ *p* < 0.001, ^****^ *p* < 0.0001.

To delineate the contribution of TREM1 to traumatic brain injury, we subjected *Trem1* ^−/−^ and wild‐type (WT) mice to the CCI model, followed by comprehensive histological and functional assessments (Figure 2a and Figure S2a). We first assessed the inflammatory response in the TBI cortex. Cytokine array and ELISA analyses showed that Trem1 deficiency markedly reduced the levels of several pro‐inflammatory mediators, including ICAM‐1, IL‐1β, CXCL1, M‐CSF, and MCP‐1, compared with WT mice (Figure 2b,c and Figure S2b). To determine whether this reduction simply reflected diminished myeloid cell infiltration, we quantified infiltrating monocyte/macrophage populations in the TBI cortex by flow cytometry at 3 days after TBI. The percentage of CD45^hi^CD11b^+^ cells was comparable between WT and *Trem1* ^−/−^ mice (Figure S2c), indicating that Trem1 deficiency did not substantially alter the extent of myeloid cell infiltration into the TBI cortex. Consistently, immunofluorescence staining revealed reduced TNF‐α signal and lower CD86 expression in the perilesional cortex of *Trem1* ^−/−^ mice (Figure 2d and Figure S2d,e). These findings suggest that Trem1 loss attenuates the inflammatory activation state of infiltrating myeloid cells rather than merely reducing their recruitment to the injured brain. We next examined the impact of TREM1 ablation on cellular survival and tissue architecture. TUNEL staining demonstrated that TREM1 deficiency significantly attenuated neuronal apoptosis in the acute phase of injury (Figure 2e and Figure S2f). Additionally, elevated levels of myelin and neuronal markers were observed in the brain cortex of *Trem1* ^−/−^ mice (Figure 2f and Figure S2g), while neuronal protein expression changes in the hippocampus were minimal (Figure 2g and Figure S2h). *Trem1* ^−/−^ mice exhibited significantly reduced brain water content, indicating alleviated cerebral edema (Figure 2h). Functional assessment of barrier permeability via Evans Blue extravasation further confirmed that TREM1 ablation minimized vascular leakage compared to the extensive disruption observed in WT mice (Figure 2i,j).

To further validate these histological findings, we analyzed cortical tissues by Western blotting. Consistent with the observed barrier and neuronal protection, *Trem1* ^−/−^ mice retained higher expression levels of the tight junction protein Occludin, as well as the synaptic markers Synaptophysin and βIII‐tubulin, whereas these proteins were markedly decreased in WT mice after TBI (Figure 2k,l).

We then asked whether these molecular and histological changes were associated with improved functional outcomes. *Trem1* ^−/−^ mice displayed superior sensorimotor coordination, with longer latencies to fall in both accelerating and fixed‐speed rotarod tests (Figure 2m,n). Neurological recovery was also improved, as indicated by lower modified neurological severity scores (mNSS) from day 1 to day 7 post‐TBI (Figure 2o). In the open field test, *Trem1* ^−/−^ mice displayed increased exploratory activity and reduced anxiety‐like behavior, as shown by representative movement tracks (Figure 2p) and a higher percentage of time spent in the center zone (Figure 2q).

After confirming the peripheral origin of brain‐infiltrating TREM1^+^ cells by GFP bone marrow transplantation, we further used head‐protected bone marrow chimeric mice to test whether peripheral hematopoietic cells could replenish the TREM1^+^ myeloid compartment after TBI. WT recipient mice were reconstituted with bone marrow from either WT or *Trem1* ^−/−^ donors before CCI, with *Trem1* ^−/−^ mice subjected to TBI serving as a genetic‐deficiency reference. Flow cytometric analysis showed that WT bone marrow transplantation into WT recipients resulted in a robust TREM1^+^CD45^+^CD11b^+^ population in the bone marrow, blood, and injured brain after TBI. In contrast, transplantation of *Trem1* ^−/−^ bone marrow into WT recipients substantially reduced the proportion of TREM1^+^CD45^+^CD11b^+^ cells across these compartments, although the frequencies remained higher than those observed in *Trem1* ^−/−^ mice after TBI (Figure S3a–c). These findings further indicate that the TREM1^+^ myeloid cells detected in the TBI cortex after TBI are largely derived from the peripheral hematopoietic compartment.

Together, these data show that TREM1 is predominantly associated with peripheral myeloid cell infiltration after TBI and contributes to neuroinflammation, vascular disruption, tissue damage, and neurological dysfunction. Genetic ablation of Trem1 limits inflammatory activation, preserves blood‐brain barrier integrity and neuronal structure, and improves behavioral recovery after CCI.

### 2.3 De Novo Design and Engineering of a TREM1‐targeting LYTAC Degrader

**FIGURE 3:**
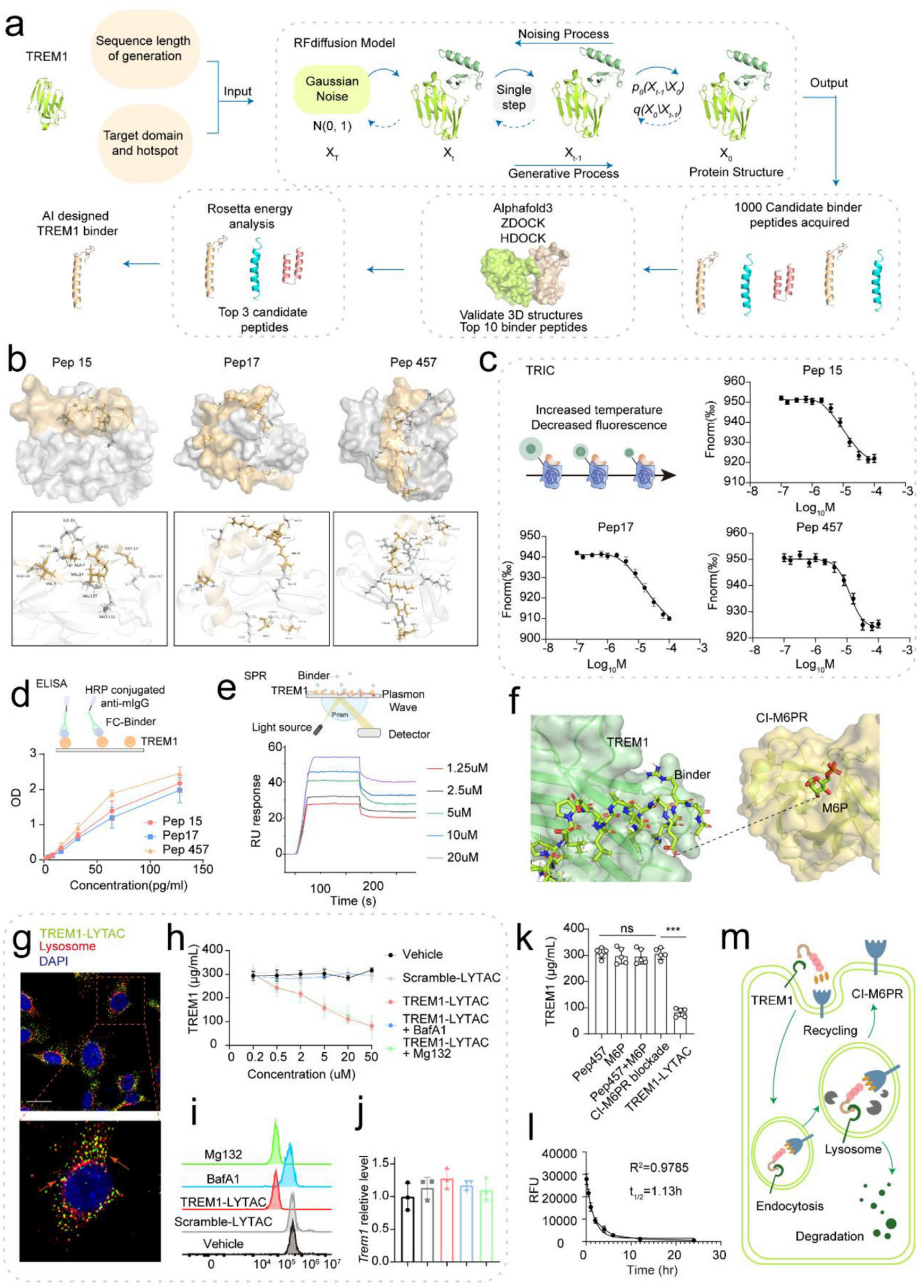
Generative AI‐driven design and mechanistic validation of a TREM1‐targeting LYTAC degrader. (a) Schematic of the RFdiffusion‐guided pipeline for de novo TREM1‐binding peptide design. Peptide backbones were generated around the Ig‐like domain of TREM1 and subsequently screened by AlphaFold3, ZDOCK, HDOCK, and Rosetta‐based energetic analysis to identify high‐ranking candidates. (b) Predicted structural models of Pep15, Pep17, and Pep457 in complex with TREM1, showing the predicted binding interfaces. (c) Temperature‐related intensity change (TRIC) assays showing concentration‐dependent target engagement of Pep15, Pep17, and Pep457 with TREM1. Left, schematic illustration of the TRIC assay principle; right, dose‐response curves of the indicated peptides (*n* = 3 independent experiments). (d) ELISA‐based binding analysis of Pep15, Pep17, and Pep457 to immobilized TREM1. Top, schematic of the ELISA workflow; bottom, quantitative binding curves (*n* = 3 independent experiments). (e) Surface plasmon resonance (SPR) analysis of Pep457 binding to TREM1. Top, schematic of the SPR assay; bottom, representative sensorgrams obtained at the indicated Pep457 concentrations. (f) Structural model of the bifunctional TREM1‐LYTAC, in which Pep457 engages TREM1 and the mannose‐6‐phosphate (M6P) moiety recruits the cation‐independent mannose‐6‐phosphate receptor (CI‐M6PR). (g) Representative confocal images showing partial colocalization of TREM1‐LYTAC with lysosomes following cellular uptake. TREM1‐LYTAC, green; lysosomes, red; nuclei, blue. Arrows indicate representative colocalized puncta. Scale bars as indicated. (h) ELISA quantification of cellular TREM1 protein following treatment with vehicle, scrambled‐LYTAC, TREM1‐LYTAC, or TREM1‐LYTAC in the presence of bafilomycin A1 (BafA1) or MG132 (*n* = 5 independent experiments). (i) Representative flow‐cytometric histograms showing cell‐surface TREM1 expression after the indicated treatments. (j) *Trem1* mRNA expression measured by qPCR after the indicated treatments (*n* = 3 independent experiments; one‐way ANOVA followed by Tukey's multiple‐comparison test). (k) Requirement of both functional modules for TREM1 reduction. Cellular TREM1 protein levels were quantified after treatment with Pep457 plus M6P, Pep457 alone, M6P alone, TREM1‐LYTAC in the presence of CI‐M6PR blockade, or conjugated TREM1‐LYTAC (*n* = 5 independent experiments; one‐way ANOVA followed by Tukey's multiple‐comparison test). (l) Plasma pharmacokinetic profile of free TREM1‐LYTAC after intravenous administration. Fluorescence intensity was fitted to a monoexponential decay model, yielding an apparent half‐life of 1.13 h (*n* = 3 mice). (m) Proposed mechanism of TREM1‐LYTAC‐mediated degradation. TREM1‐LYTAC bridges cell‐surface TREM1 to CI‐M6PR, thereby promoting receptor internalization and lysosomal degradation. Data are mean ± SD. Each dot represents an independent experimental replicate or an individual animal. Statistical tests are specified for each quantitative panel above. Exact *p* values are provided in the Source Data. ^*^ *p* < 0.05, ^**^ *p* < 0.01, ^***^ *p* < 0.001, ^****^ *p* < 0.0001.

Current therapeutic strategies for TREM1, such as the LP17 decoy peptide, rely on passive sequestration of ligands, which often yields suboptimal suppression of receptor activation. To achieve potent and direct inhibition, we employed a generative deep‐learning approach to design high‐affinity TREM1 binders (Figure 3a). Using the RFdiffusion model, we conditioned the generation process on the Ig‐like domain of mouse TREM1 (residues 24–124) to generate de novo peptide backbones (20–50 residues) capable of high‐affinity binding. A total of 1000 candidate scaffolds were subsequently evaluated by AlphaFold3, ZDOCK, HDOCK, and Rosetta‐based energetic filtering. This workflow identified three top‐ranking candidates, Pep15, Pep17, and Pep457, which were predicted to engage the ligand‐binding hotspot of mouse TREM1 with high structural complementarity (Figure 3b).

We next experimentally evaluated peptide binding to mouse TREM1. Temperature‐related intensity change (TRIC) assays showed concentration‐dependent fluorescence changes for all three candidates, consistent with target engagement (Figure 3c). ELISA‐based binding analysis further identified Pep457 as the strongest binder among the candidates tested (Figure 3d), and SPR confirmed direct Pep457–TREM1 binding with measurable association and dissociation kinetics (Figure 3e). Based on these computational and experimental results, Pep457 was selected as the lead TREM1‐binding peptide.

To assess whether Pep457 might be compatible with human TREM1, we modeled the interaction between Pep457 and the human TREM1 ectodomain using AlphaFold‐based structural prediction. The predicted complex placed Pep457 within a structurally compatible surface pocket of the human TREM1 Ig‐like domain and suggested multiple potential polar contacts at the binding interface (Figure S4). This predicted binding mode was broadly similar to that observed in the mouse TREM1–Pep457 model, suggesting that Pep457 may retain structural compatibility with human TREM1 and providing a rationale for future experimental validation of cross‐species binding.

To enable active receptor elimination rather than receptor blockade alone, Pep457 was chemically conjugated to mannose‐6‐phosphate (M6P) to generate a TREM1‐targeting lysosome‐targeting chimera (TREM1‐LYTAC) designed to recruit the cation‐independent mannose‐6‐phosphate receptor (CI‐M6PR) (Figure 3f). Binding analysis showed that M6P conjugation did not markedly compromise Pep457 binding to mouse TREM1, as the TREM1‐LYTAC retained a binding profile comparable to that of unconjugated Pep457 (Figure S5).

We then evaluated whether TREM1‐LYTAC reduced TREM1 protein abundance in cells. Immunofluorescence imaging showed that TREM1‐LYTAC signals partially colocalized with lysosomal staining, suggesting that the chimera was delivered to lysosome‐associated compartments after cellular uptake (Figure 3g). We then quantified TREM1 abundance after TREM1‐LYTAC treatment. ELISA analysis further showed that TREM1‐LYTAC reduced cellular TREM1 protein levels in a dose‐dependent manner, whereas vehicle and scrambled‐LYTAC controls had little effect; this reduction was largely prevented by the lysosomal inhibitor bafilomycin A1 but not by the proteasome inhibitor MG132 (Figure 3h). In parallel, flow cytometry confirmed a decrease in cell‐surface TREM1 after TREM1‐LYTAC treatment, which was similarly rescued by bafilomycin A1 but not by MG132 (Figure 3i). By contrast, qPCR analysis showed no significant decrease in Trem1 mRNA levels across treatment groups, indicating that TREM1‐LYTAC reduced TREM1 abundance mainly at the protein level rather than by suppressing Trem1 transcription (Figure 3j). Together, these results support a predominantly lysosome‐dependent mechanism for TREM1‐LYTAC‐mediated reduction of TREM1 protein.

We further tested whether both functional modules of TREM1‐LYTAC were required for TREM1 reduction. Pep457 alone, M6P alone, or an unconjugated mixture of Pep457 and M6P did not reduce TREM1 protein to the same extent as the conjugated TREM1‐LYTAC. In addition, blockade of CI‐M6PR attenuated the TREM1‐LYTAC‐induced decrease in TREM1 protein (Figure 3k). These findings indicate that efficient TREM1 degradation requires both the TREM1‐binding Pep457 moiety and the M6P‐dependent lysosomal trafficking arm. To characterize the systemic exposure profile of TREM1‐LYTAC, we next assessed its plasma pharmacokinetics. Plasma fluorescence signals declined rapidly over time and were well fitted by a monoexponential decay model, yielding an apparent plasma half‐life of approximately 1.13 h (Figure 3l).

Together, these findings identify Pep457 as a de novo designed TREM1‐binding peptide and show that its M6P‐conjugated LYTAC derivative reduces TREM1 protein through a predominantly lysosome‐dependent mechanism (Figure 3m).

### 2.4 Engineering and Validation of a BBB‐Targeted, pH‐Responsive Nanodevice for Spatiotemporal Delivery of TREM1‐LYTAC

**FIGURE 4:**
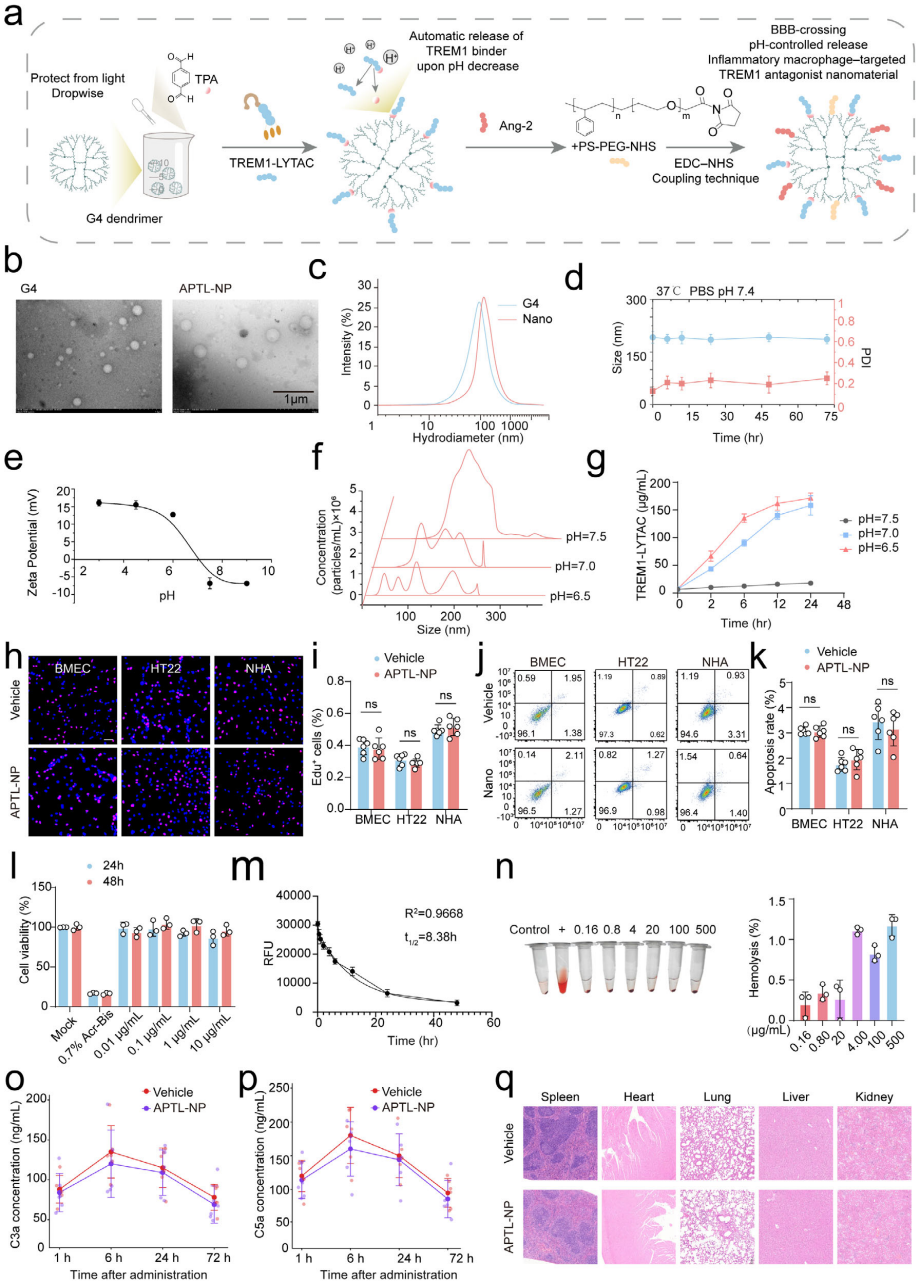
Engineering and validation of a BBB‐targeted, pH‐responsive nanodevice for spatiotemporal delivery of TREM1‐LYTAC. (a) Schematic illustration of APTL‐NP fabrication. TREM1‐LYTAC was conjugated to a generation‐4 PAMAM dendrimer through a terephthalaldehyde‐derived acid‐labile linker, followed by surface functionalization with Angiopep‐2 and PS‐PEG‐NHS to enable LRP1‐directed BBB targeting and improve systemic stability. (b) Representative transmission electron microscopy images of PAMAM‐based precursor particles and APTL‐NP (Scale bars, 1 µm). (c) Dynamic light scattering profiles showing the apparent hydrodynamic size distributions of G4 PAMAM and APTL‐NP under the indicated measurement conditions. (d) Time‐dependent changes in hydrodynamic diameter and polydispersity index of APTL‐NP incubated in PBS at pH 7.4 and 37°C. (e) pH‐dependent zeta‐potential profile of APTL‐NP. (f) Nanoparticle tracking analysis showing pH‐dependent particle size and concentration distributions after incubation at pH 7.5, 7.0, or 6.5. (g) pH‐responsive release of TREM1‐binding‐competent TREM1‐LYTAC from APTL‐NP. Released free TREM1‐LYTAC was separated by ultrafiltration and quantified using a TREM1‐binding ELISA with free TREM1‐LYTAC standards (*n* = 3 independent experiments). (h) Representative EdU staining images of BMECs, HT22 cells, and NHAs treated with vehicle or APTL‐NP. (i) Quantification of EdU‐positive cells in the indicated cell types(*n* = 6 independent experiments, two‐way ANOVA followed by Tukey's multiple‐comparison test). (j) Representative flow‐cytometric plots for apoptosis analysis in BMECs, HT22 cells, and NHAs after treatment with vehicle or APTL‐NP. (k) Quantification of apoptotic cells in the indicated cell types(*n* = 6 independent experiments, two‐way ANOVA followed by Tukey's multiple‐comparison test). (l) Cell viability after 24 h or 48 h exposure to the indicated concentrations of APTL‐NP (*n* = 3 independent experiments, two‐way ANOVA followed by Tukey's multiple‐comparison test). (m) Plasma pharmacokinetic profile of APTL‐NP after intravenous administration. The fluorescence signal was fitted to a monoexponential decay model, yielding an apparent half‐life of 8.38 h (*n* = 3 mice). (n) Representative photographs and quantification of hemolysis induced by the indicated concentrations of APTL‐NP (*n* = 3 independent experiments, one‐way ANOVA followed by Tukey's multiple‐comparison test). (o, p) Plasma C3a and C5a concentrations at the indicated time points after administration of vehicle or APTL‐NP (*n* = 6 mice per group, Two‐way ANOVA followed by Tukey's multiple‐comparison test). (q) Representative H&E‐stained sections of the spleen, heart, lung, liver, and kidney from vehicle‐ and APTL‐NP‐treated mice (Scale bar, 100 µm). Data are presented as mean ± SD where applicable. For in vitro assays, n denotes independent experimental replicates; for in vivo analyses, n denotes individual mice. Statistical tests are specified for each quantitative panel above. Exact *p* values are provided in the Source Data. ns, not significant; ^*^ *p* < 0.05, ^**^ *p* < 0.01, ^***^ *p* < 0.001, ^****^ *p* < 0.0001.

Although free TREM1‐LYTAC efficiently reduced TREM1 abundance in vitro, its rapid systemic elimination and limited brain delivery may restrict its therapeutic application after TBI. To improve systemic persistence and enable lesion‐responsive payload delivery, we constructed a multifunctional nanodevice based on a generation‐4 poly(amidoamine) (G4 PAMAM) dendrimer core (Figure 4a). Angiopep‐2 was introduced to provide low‐density lipoprotein receptor‐related protein‐1 (LRP1)‐directed BBB‐targeting functionality, whereas TREM1‐LYTAC was conjugated to the dendrimer through a terephthalaldehyde‐derived acid‐labile Schiff‐base linker designed to permit pH‐responsive release. The resulting construct was further stabilized with PS‐PEG‐NHS to improve colloidal stability and systemic residence.

The chemical functionalization of the resulting nanodevice was characterized by ^1^H NMR spectroscopy (Figure S6a). The appearance of characteristic resonances attributable to the PAMAM scaffold and the conjugated components supported successful multicomponent functionalization of the nanoplatform. The final formulation was termed Angiopep‐2/PAMAM/TPA/LYTAC nanoparticle (APTL‐NP).

Transmission electron microscopy revealed that APTL‐NP formed well‐dispersed, near‐spherical nanostructures. Dynamic light scattering showed an apparent hydrodynamic diameter of approximately 180 nm for APTL‐NP. Given that this dimension is substantially larger than the expected molecular size of an individual G4 PAMAM dendrimer, the measured particle population is more appropriately interpreted as higher‐order PAMAM‐based assemblies rather than individual modified dendrimer molecules. Importantly, the particle size and polydispersity index remained stable during incubation in PBS at pH 7.4 for up to 72 h. APTL‐NP also retained stable physicochemical properties in 50% fetal bovine serum at 37°C (Figure S6b), supporting the colloidal stability of the assembled nanoparticle population under the tested conditions. We next determined whether the pH conditions used for in vitro release studies were relevant to the TBI microenvironment. Direct measurement of lesion pH showed that the injured brain became acidic during the acute and subacute phases after TBI. Compared with sham animals, lesion pH decreased during the first 1–3 days after injury and subsequently recovered toward baseline by day 7 (Figure S7). These findings establish the presence of a transient acidic lesion microenvironment after TBI. Notably, the pH 7.0 condition used in the release assay closely approximated the measured lesion pH range, whereas pH 6.5 was included as a more acidic boundary condition. Consistent with the acid‐labile design, APTL‐NP exhibited pH‐dependent changes in surface charge, including a marked transition in zeta potential across the tested pH range (Figure 4e). Particle‐size distribution analysis further revealed pronounced changes in the nanoparticle population under mildly acidic conditions relative to near‐physiological pH (Figure 4f), consistent with pH‐triggered structural reorganization of the nanodevice. To directly assess pH‐responsive payload release, APTL‐NP was incubated in PBS at pH 7.5, 7.0, or 6.5. At the indicated time points, free TREM1‐LYTAC was separated from intact nanoparticles by ultrafiltration and quantified using a TREM1‐binding ELISA with free TREM1‐LYTAC standards (Figure 4g). This assay measured released payload that retained TREM1‐binding activity.

APTL‐NP showed minimal TREM1‐LYTAC release at pH 7.5, whereas acidic conditions markedly increased release over time. Release was more rapid at pH 6.5 during the early phase, and both pH 6.5 and pH 7.0 resulted in substantially greater release than pH 7.5. These data demonstrate that acidic conditions promote the release of TREM1‐binding‐competent LYTAC from APTL‐NP, supporting its pH‐responsive behavior in the acidic TBI lesion microenvironment.

Given the potential biocompatibility concerns associated with cationic dendrimer‐based materials, we evaluated the safety profile of APTL‐NP. In brain microvascular endothelial cells (BMECs), hippocampal neurons (HT22), and normal human astrocytes (NHAs), APTL‐NP did not significantly affect cell proliferation, as assessed by EdU incorporation (Figure 4h,i), or increase apoptosis relative to vehicle‐treated controls (Figure 4j,k). Cell viability remained preserved across the tested concentration range at both 24 and 48 h (Figure 4l).

We next assessed whether nanoformulation improved the systemic exposure profile of TREM1‐LYTAC. Following intravenous administration, APTL‐NP exhibited an apparent plasma half‐life of approximately 8.38 h (Figure 4m), indicating prolonged systemic persistence compared with free TREM1‐LYTAC. In addition, APTL‐NP caused negligible hemolysis even at the high tested concentration (Figure 4n), supporting its blood compatibility.

To further assess systemic compatibility, plasma C3a and C5a levels were monitored following APTL‐NP administration. Neither marker was significantly elevated compared with vehicle controls (Figure 4o,p), indicating that APTL‐NP did not induce detectable acute complement activation. Although TBI itself increased circulating C3a and C5a levels, APTL‐NP did not further exacerbate this injury‐associated complement response (Figure S8a,b). In parallel, APTL‐NP did not cause detectable reductions in circulating leukocyte subsets or alterations in serum hepatic and renal biochemical indices (Figure S8c–j). Histological examination of the spleen, heart, lung, liver, and kidney likewise revealed no overt treatment‐related abnormalities (Figure 4q). Together, these data support the favorable acute systemic compatibility of APTL‐NP and argue against treatment‐associated complement overactivation, leukocyte depletion, or overt systemic toxicity under the tested regimen.

Collectively, these data establish APTL‐NP as a stable and blood‐compatible nanoplatform that enables pH‐responsive release of TREM1‐binding‐competent TREM1‐LYTAC and prolongs systemic exposure. Under the tested dose and observation period, APTL‐NP was not associated with detectable complement overactivation, leukocyte depletion, hematological abnormalities, or overt organ toxicity. These findings support the acute systemic compatibility of APTL‐NP, while direct evaluation of antimicrobial host‐defense function will be required to fully exclude potential effects of peripheral TREM1 suppression on infection susceptibility.

### 2.5 APTL‐NP Enhances BBB‐Associated Delivery and Preferentially Accumulates in Myeloid Cells Within the Injured Brain

**FIGURE 5:**
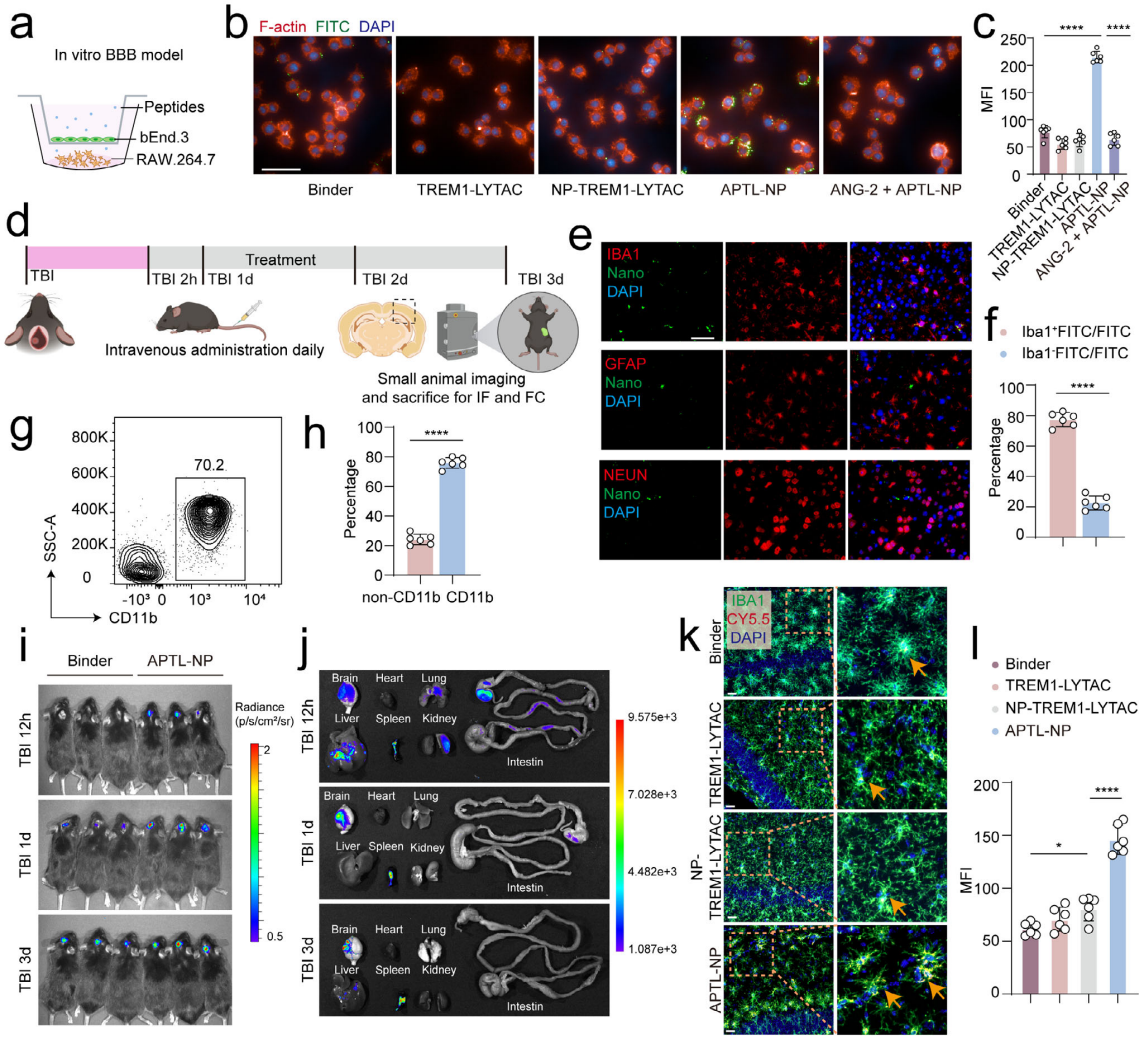
APTL‐NP enhances BBB‐associated delivery and preferentially accumulates in inflammatory myeloid cells after TBI. (a) Schematic of the in vitro blood‐brain barrier model, consisting of a bEnd.3 endothelial monolayer in the upper chamber and RAW264.7 macrophages in the lower chamber. (b, c) In vitro assessment of BBB‐associated transport. (b) Representative fluorescence images of RAW264.7 cells in the lower chamber after apical administration of FITC‐labeled Binder, TREM1‐LYTAC, NP‐TREM1‐LYTAC, APTL‐NP, or Angiopep‐2‐preblocked APTL‐NP (scale bar, 50 µm). (c) Quantification of mean fluorescence intensity in RAW264.7 cells (*n* = 6; ne‐way ANOVA with Tukey's multiple‐comparison test). (d) Schematic timeline of the in vivo study. Mice received intravenous injections of free binder or APTL‐NP at 2 h, 1 day, and 2 days post‐TBI, followed by imaging and tissue analysis at 3 days post‐TBI. (e, f) Immunofluorescence analysis of APTL‐NP cellular distribution in the injured brain. (e) Representative images showing FITC‐labeled APTL‐NP signal relative to IBA1^+^ myeloid cells, GFAP^+^ astrocytes, or NeuN^+^ neurons in the perilesional cortex. FITC‐labeled APTL‐NP is shown in green, cellular markers in red, and nuclei in blue. (f) Quantification of APTL‐NP‐associated fluorescence colocalized with IBA1^+^ vs. IBA1^−^ cells (*n* = 6 mice; scale bar, 50 µm, two‐tailed paired Student's *t*‐test). (g, h) Flow‐cytometric validation of myeloid‐cell uptake in dissociated injured brain tissue. (g) Representative contour plot identifying APTL‐NP‐positive cells. (h) Quantification of the fraction of APTL‐NP‐positive cells expressing CD11b (*n* = 6 mice; two‐tailed paired Student's *t*‐test). (i) Longitudinal in vivo fluorescence imaging of mice receiving free binder or APTL‐NP after TBI. (j) Ex vivo fluorescence imaging of brain and major peripheral organs harvested at the indicated time points after TBI. (k, l) In vivo comparison of brain delivery among Cy5.5‐labeled Binder, TREM1‐LYTAC, NP‐TREM1‐LYTAC lacking Angiopep‐2, and APTL‐NP. k, Representative confocal images of the perilesional cortex showing Cy5.5‐associated fluorescence (red) relative to IBA1‐positive myeloid cells (green); nuclei are counterstained with DAPI (blue). Dashed boxes indicate regions shown at higher magnification, and arrows indicate Cy5.5‐associated fluorescence within or adjacent to IBA1‐positive cells (Scale bar, 50 µm). l, Quantification of Cy5.5 mean fluorescence intensity in the perilesional cortex (*n* = 6 mice per group; one‐way ANOVA followed by Tukey's multiple‐comparison test). Data are presented as mean ± SD. Each dot represents an independent Transwell experiment in (c) or an individual animal in (f), (h), and (l). For multi‐group comparisons in (c) and (l), statistical significance was assessed by one‐way ANOVA followed by Tukey's multiple‐comparison test. For pairwise comparisons in (f) and (h), paired two‐tailed Student's *t*‐test was used. Statistical comparisons are indicated by brackets in the corresponding panels. ^*^ *p* < 0.05, ^**^ *p* < 0.01, ^***^ *p* < 0.001, and ^****^ *p* < 0.0001.

To evaluate whether APTL‐NP facilitates Angiopep‐2/LRP1‐mediated BBB transport and subsequent delivery to TBI‐associated inflammatory cells, we established an in vitro BBB–macrophage co‐culture model consisting of a bEnd.3 endothelial monolayer and RAW264.7 macrophages in the lower chamber (Figure 5a). After apical administration, APTL‐NP exhibited markedly higher accumulation in RAW264.7 cells than the free binder, non‐Angiopep‐2 nanoparticles, and the Angiopep‐2 preblocking group, as shown by fluorescence imaging and MFI quantification (Figure 5b,c). These findings indicate that Angiopep‐2/LRP1‐associated transport contributes to the enhanced BBB delivery of APTL‐NP, in addition to the improved systemic exposure conferred by nanoformulation. In parallel, TEER values remained stable and comparable among all groups over 24 h, indicating that APTL‐NP promoted trans‐BBB delivery without overtly disrupting endothelial barrier integrity (Figure S9).

We next assessed the in vivo biodistribution and cellular tropism of APTL‐NP in the CCI mouse model (Figure 5d). Immunofluorescence analysis of brain sections showed that APTL‐NP‐associated fluorescence was enriched in the perilesional region and largely colocalized with IBA1^+^ myeloid cells, whereas limited overlap was observed with GFAP^+^ astrocytes or NeuN^+^ neurons (Figure 5e). Quantitative analysis showed that approximately 80% of APTL‐NP‐associated fluorescence overlapped with IBA1^+^ cells (Figure 5f). Flow cytometric analysis of TBI brain homogenates further showed that most APTL‐NP‐positive cells expressed CD11b, supporting preferential uptake by brain‐infiltrating and resident myeloid cells after TBI (Figure 5g,h).

We further examined whole‐body biodistribution and retention of APTL‐NP. Longitudinal IVIS imaging showed that, whereas the free binder signal declined rapidly, APTL‐NP produced a stronger and more persistent fluorescence signal in the head region from 12 h to 1 day after injury (Figure 5i and Figure S10a). Ex vivo imaging of major organs confirmed the expected hepatic and splenic distribution, while fluorescence intensity in the TBI brain was higher in APTL‐NP‐treated mice than in mice receiving the free binder (Figure 5j and Figure S10b). To further distinguish the contribution of nanoformulation from that of Angiopep‐2‐mediated BBB transport in vivo, we compared free Binder, TREM1‐LYTAC, NP‐TREM1‐LYTAC lacking Angiopep‐2, and APTL‐NP under identical administration conditions. Confocal imaging of the perilesional cortex showed a modest increase in brain‐associated fluorescence following nanoformulation, whereas Angiopep‐2‐functionalized APTL‐NP exhibited markedly greater accumulation than NP‐TREM1‐LYTAC (Figure 5k,l). These findings indicate that nanoformulation improves systemic exposure and contributes to brain delivery, while Angiopep‐2 provides an additional enhancement of BBB‐associated transport in vivo. Collectively, these data indicate that APTL‐NP improves BBB‐associated delivery, increases accumulation in the injured brain, and preferentially targets myeloid‐lineage cells within the post‐TBI inflammatory microenvironment.

### 2.6 APTL‐NP‐Mediated TREM1 Targeting Attenuates Neuroinflammation and Promotes Neuroprotection In Vivo

**FIGURE 6:**
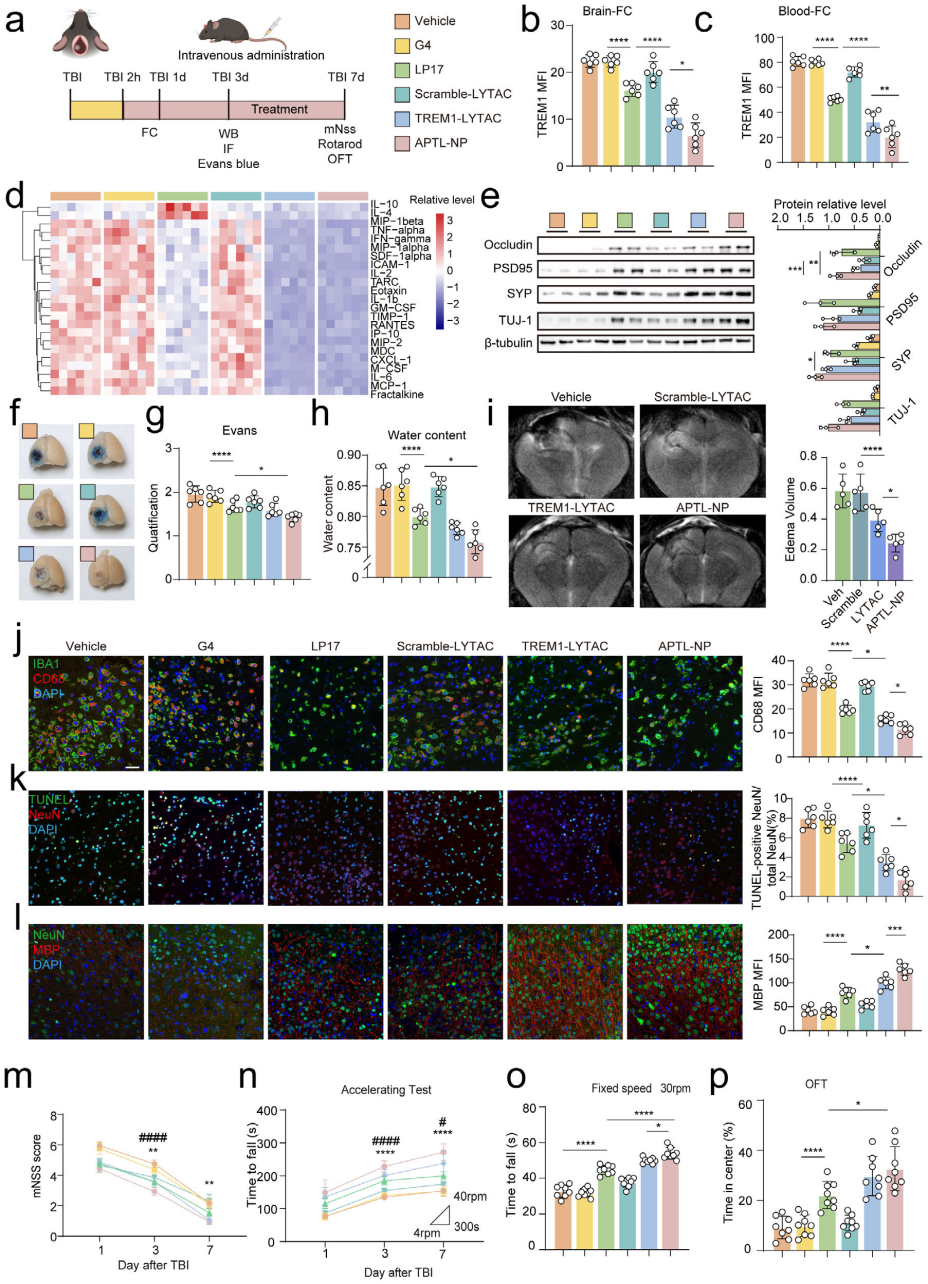
APTL‐NP reduces TREM1 abundance, attenuates neuroinflammation, and promotes neurovascular protection and functional recovery after TBI. (a) Schematic timeline of the therapeutic regimen. Mice received intravenous administrations of the APTL‐NP at 2 h, 1 day, and 2 days post‐TBI. (b, c) Flow cytometric quantification of TREM1 surface expression in (b) dissociated brain tissue and (c) peripheral blood at 3 days post‐TBI. The APTL‐NP treatment induced the strongest reduction in TREM1 surface signal (*n* = 6 mice per group; one‐way ANOVA). (d) Heatmap visualization of the cortical cytokine/chemokine profile. Systemic administration of the APTL‐NP effectively attenuated the TBI‐induced pro‐inflammatory signature, reducing levels of key mediators while sparing anti‐inflammatory factors. (e) Western blot analysis of blood‐brain barrier (BBB) and synaptic structural proteins (Occludin, PSD95, Synaptophysin/SYP, TUJ‐1) in the perilesional cortex. Densitometric quantification (right) confirms the rescue of structural integrity by the APTL‐NP treatment (*n* = 3 independent experiments; two‐way ANOVA). (f–h) Assessment of BBB permeability and cerebral edema. (f) Representative images of whole brains following Evans Blue perfusion. (g) Quantification of Evans Blue extravasation (*n* = 6 mice per group; one‐way ANOVA). (h) Brain water content analysis indicating significant alleviation of cerebral edema in the APTL‐NP group (*n* = 6 mice per group; one‐way ANOVA). (i) T2‐weighted (T2W) images of the mouse coronal hippocampal layer in different treatment groups after TBI (3d) and quantitation of edema volume (*n* = 5 mice per group, one‐way ANOVA). (j) Immunofluorescence analysis of microglial activation. Representative images of Iba1 (green) and the lysosomal activation marker CD68 (red). The APTL‐NP treatment significantly suppressed the expansion of CD68^+^ activated macrophages (*n* = 6 mice per group, one‐way ANOVA, scale bar, 50 µm). (k) Evaluation of neuronal apoptosis. Representative staining for TUNEL (green) and NeuN (red) in the pericontusional cortex, showing reduced neuronal death in treated mice (*n* = 6 mice per group, one‐way ANOVA, scale bar, 50 µm). (l) Assessment of white matter integrity. Representative images of Myelin Basic Protein (MBP, red) and NeuN (green), demonstrating preservation of myelin structure (*n* = 6, one‐way ANOVA, scale bar, 50 µm). (m–p) Longitudinal assessment of sensorimotor and exploratory behavioral performance (*n* = 8 mice per group). (m) Neurological impairment assessed by the Modified Neurological Severity Score (mNSS) (two‐way ANOVA, ^*^ indicate comparisons between LP17 and APTL‐NP, whereas ^#^ indicate comparisons between TREM1‐LYTAC and APTL‐NP). (n) Motor coordination evaluated by the accelerating Rotarod test (two‐way ANOVA, ^*^ indicate comparisons between LP17 and APTL‐NP, whereas ^#^ indicate comparisons between TREM1‐LYTAC and APTL‐NP). (o) Motor performance assessed using the fixed‐speed rotarod test at 30 rpm (one‐way ANOVA). (p) Anxiety‐like behavior assessed by the time spent in the center zone of the Open Field Test (OFT) (one‐way ANOVA). Data are presented as mean ± SD. Each dot represents an independent animal unless otherwise indicated. Statistical significance was assessed by one‐way ANOVA followed by Tukey's multiple‐comparison test for (b), (c), (e), (g–l), (o), and (p), and by two‐way ANOVA followed by Tukey's multiple‐comparison test for the longitudinal analyses in m and n. Exact *p* values are provided in the Source Data. ^*^ *p* < 0.05, ^**^ *p* < 0.01, ^***^ *p* < 0.001, ^****^ *p* < 0.0001, ^#^ *p* < 0.05, ^##^ *p* < 0.01, ^###^ *p* < 0.001, ^####^ *p* < 0.0001.

To evaluate the therapeutic efficacy of APTL‐NP in vivo, mice were subjected to CCI and treated according to the indicated experimental scheme (Figure 6a). We first assessed the overall therapeutic effect of APTL‐NP relative to both injured and uninjured reference conditions. Comparison of Sham + Vehicle, TBI + Vehicle, and TBI + APTL‐NP groups showed that APTL‐NP substantially ameliorated TBI‐associated neuroinflammation, blood‐brain barrier dysfunction, cerebral edema, and neurological deficits, with multiple outcome measures shifting toward sham levels (Figure S11). We next compared the individual therapeutic formulations to further delineate the contribution of TREM1 targeting and nanoformulation. Flow‐cytometric analysis showed that APTL‐NP produced the greatest reduction in TREM1 abundance in both the TBI brain and peripheral blood, whereas free TREM1‐LYTAC and LP17 showed more limited effects (Figure 6b,c). These findings support enhanced in vivo target engagement by the APTL‐NP formulation.

Multiplex profiling of TBI cortical tissue showed that APTL‐NP broadly attenuated the TBI‐associated inflammatory cytokine and chemokine response (Figure 6d). Consistent with reduced neuroinflammation, APTL‐NP preserved the expression of the tight‐junction protein Occludin, as well as the neuronal and synaptic proteins PSD95, SYP, and TUJ‐1 (Figure 6e). Functionally, APTL‐NP reduced Evans blue extravasation and brain water content, indicating improved blood–brain barrier integrity and reduced cerebral edema (Figure 6f–h). T2‐weighted magnetic resonance imaging (MRI) further confirmed reduced edema volume in APTL‐NP‐treated mice (Figure 6i).

At the cellular level, APTL‐NP reduced myeloid activation in the TBI cortex, as reflected by decreased CD68 immunoreactivity, and lowered the proportion of TUNEL‐positive NeuN‐positive neurons (Figure 6j,k). In parallel, APTL‐NP preserved myelin integrity, as indicated by increased myelin basic protein (MBP) fluorescence intensity (Figure 6l). These histopathological improvements were accompanied by improved neurological recovery. Compared with control formulations, APTL‐NP‐treated mice showed lower modified neurological severity scores, improved performance in accelerating and fixed‐speed rotarod tests, and increased center‐zone exploration in the open‐field test (Figure 6m–p).

To determine whether the therapeutic effects of APTL‐NP were dependent on TREM1 rather than nonspecific effects of the nanocarrier, selected endpoints were further evaluated in Trem1‐deficient mice. In this setting, APTL‐NP did not confer additional reductions in Evans blue leakage or brain water content relative to control formulations (Figure S12a–c). Likewise, APTL‐NP did not further improve accelerating rotarod performance or modified neurological severity scores in Trem1‐deficient mice (Figure S12d,e). Thus, the loss of incremental efficacy in the absence of TREM1 supports that the therapeutic benefit of APTL‐NP is predominantly TREM1‐dependent rather than attributable to nonspecific effects of the dendrimer‐based delivery platform.

Collectively, these findings demonstrate that APTL‐NP reduces TREM1 abundance in vivo, attenuates post‐traumatic neuroinflammation, preserves neurovascular and neuronal integrity, and improves neurological recovery through a predominantly TREM1‐dependent mechanism.

### 2.7 Single‐Cell Profiling Suggests Coordinated Remodeling of the Post‐TBI Neuroimmune Microenvironment After APTL‐NP Treatment

**FIGURE 7:**
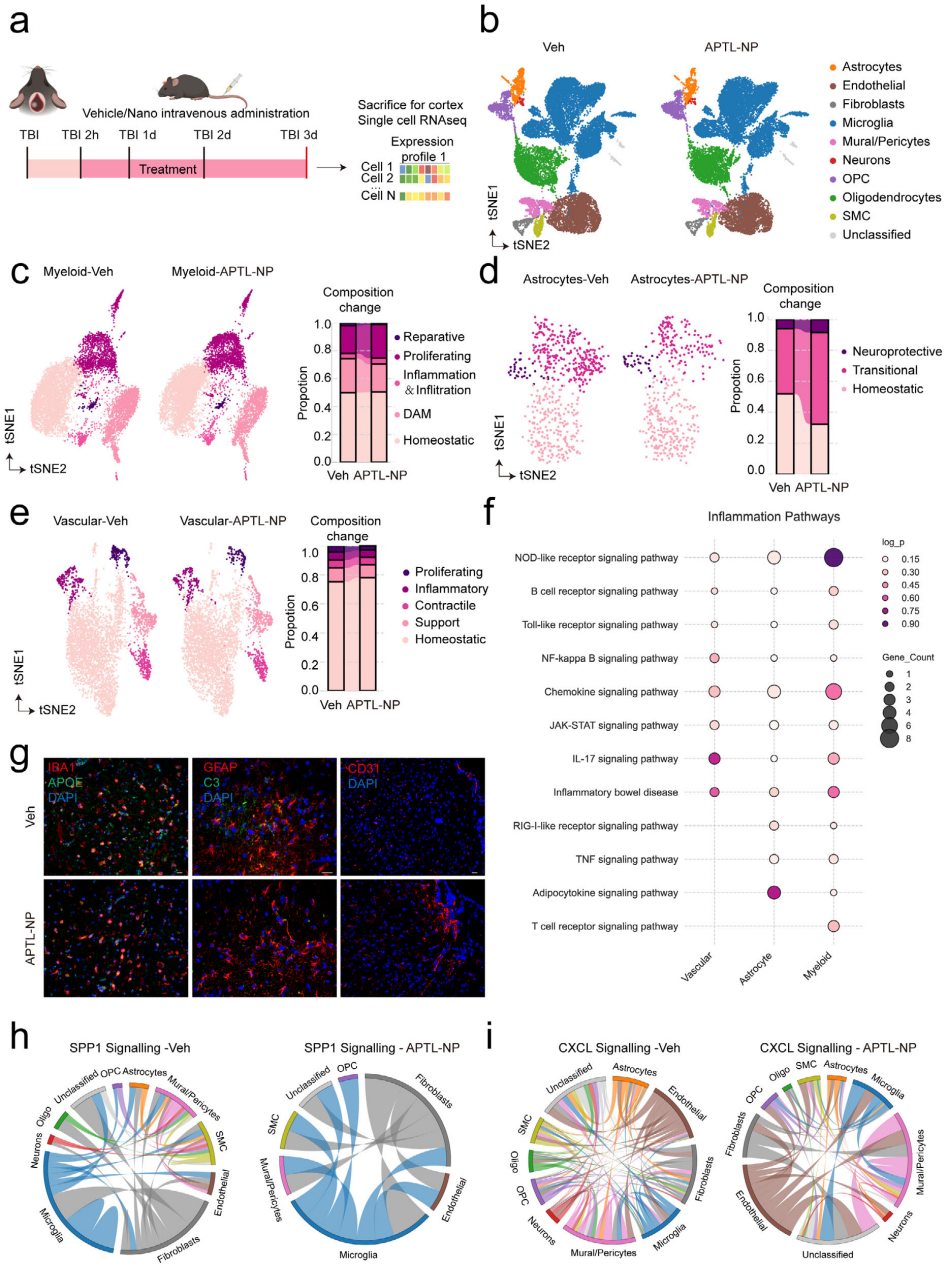
Single‐cell transcriptomics suggests coordinated remodeling of myeloid, astrocytic, vascular, and inferred intercellular communication programs after APTL‐NP treatment. (a) Schematic workflow for single‐cell RNA‐sequencing analysis of ipsilateral cortical tissue collected at 3 days after TBI from vehicle‐ and APTL‐NP‐treated mice. For this discovery dataset, cortices from three mice per group were pooled before library preparation; therefore, comparisons of cell‐state abundance and inferred cell–cell communication are presented as treatment‐associated descriptive patterns rather than independent animal‐level statistical inferences. (b) t‐SNE visualization of major cortical cell populations identified after quality control, integration, clustering, and cell‐type annotation. (c) Subclustering analysis of myeloid cells. Relative abundance and marker‐gene expression patterns are shown for infiltrating macrophages, disease‐associated microglia‐like cells, reparative myeloid cells, and other myeloid subpopulations in vehicle‐ and APTL‐NP‐treated groups. (d) Subclustering analysis of astrocytes, showing treatment‐associated shifts among reactive, transitional, homeostatic‐like, and putative protective astrocytic states. (e) Subclustering analysis of vascular‐associated cells, showing treatment‐associated changes in endothelial and fibroblast‐like populations, including Cldn5^high^/Ocln^high^ endothelial cells and Col1a1^high^/Dcn^high^ fibrotic‐like subpopulations. (f) Pathway‐level analysis of treatment‐associated transcriptional programs across myeloid, astrocytic, and vascular compartments. Enriched pathways include inflammatory and immune signaling modules such as NOD‐like receptor, Toll‐like receptor, NF‐κB, chemokine, JAK–STAT, and IL‐17 signaling. (g) Immunofluorescence validation of selected cell‐state markers in the injured cortex. Representative images show IBA1 and APOE, GFAP and C3, and CD31 staining in vehicle‐ and APTL‐NP‐treated mice. Nuclei are shown in blue. Scale bars, as indicated. (h and i) CellChat‐based inference of ligand–receptor communication networks. (h) Inferred SPP1‐associated communication in vehicle‐ and APTL‐NP‐treated groups. (i) Inferred CXCL‐associated communication in vehicle‐ and APTL‐NP‐treated groups. These analyses nominate SPP1‐ and CXCL‐associated signaling as candidate pathways linked to treatment‐associated remodeling of the post‐traumatic microenvironment.

To characterize cellular programs associated with APTL‐NP treatment after TBI, we performed single‐cell RNA sequencing of ipsilateral cortical tissue collected 3 days after injury from vehicle‐ and APTL‐NP‐treated mice (Figure 7a). For this discovery dataset, ipsilateral cortices from three mice per group were pooled before library preparation. Accordingly, comparisons of cell‐state abundance and CellChat output were interpreted as treatment‐associated descriptive patterns rather than independent animal‐level statistical inferences. Major cortical cell populations, including myeloid cells, astrocytes, endothelial cells, neurons, oligodendrocytes, and fibroblast‐like cells, were identified based on canonical marker expression (Figure 7b and Figure S13a). To further explore cellular heterogeneity, we conducted sub‐clustering analysis on key cell populations.

Sub‐clustering of the myeloid compartment revealed five distinct populations, including infiltrating macrophages defined by high expression of *Ly6c2* and *Ccl2* [29], disease‐associated microglia (DAM)‐like cells bearing the canonical Apoe and Spp1 signature [30], and a reparative subset enriched for *Arg1* and *Mrc1* [31] (Figure S13b). The Apoe/Spp1‐associated DAM‐like population showed a modest reduction in relative abundance after APTL‐NP treatment, decreasing from 24.37% in the Vehicle group to 20.01% in the APTL‐NP group. Given the pooled design, this observation is interpreted as a treatment‐associated shift in myeloid composition and does not, by itself, distinguish reduced myeloid recruitment from direct state conversion (Figure 7c). Similarly, astrocytes were sub‐clustered into reactive states high in *C3* and Serping1 and putative neuroprotective states enriched in *S100a10* and *Ptx3* (Figure S13c) [32]. APTL‐NP treatment was associated with a redistribution of astrocyte states, with changes involving both homeostatic‐like and transitional populations. These shifts are best interpreted as treatment‐associated remodeling of the TBI‐associated astrocytic response rather than as evidence for a simple restoration or loss of astrocyte homeostasis (Figure 7d). In the vascular compartment, APTL‐NP administration was associated with a decrease in *Col1a1* high/*Dcn* high fibrotic subpopulations and the preservation of *Cldn5* high/*Ocln* high endothelial cells indicative of BBB support (Figure 7e and Figure S13d) [33], This transcriptional pattern was consistent with the improved vascular integrity observed in the injured brain following APTL‐NP treatment.

Differential pathway analysis across vascular, astrocytic, and myeloid populations identified inflammation‐related signaling modules, including NOD‐like receptor, Toll‐like receptor, NF‐κB, chemokine, JAK–STAT, and IL‐17 pathways, as major treatment‐associated transcriptional programs (Figure 7f). These findings suggest that APTL‐NP is associated with broad attenuation of inflammatory signaling across multiple cellular compartments.

Immunofluorescence analysis provided orthogonal support for the single‐cell observations. Compared with vehicle treatment, APTL‐NP reduced the abundance of IBA1^+^APOE^+^ activated myeloid cells and GFAP^+^C3^+^ reactive astrocytes, while preserving CD31^+^ vascular structures in the injured cortex (Figure 7g). These results support an overall reduction in inflammatory activation together with improved vascular preservation.

To explore potential changes in intercellular signaling, CellChat analysis was used to infer ligand‐receptor communication networks. In the Vehicle group, the predicted SPP1 signaling network showed dense interactions involving microglia and fibroblast‐like cells; these inferred interactions were reduced after APTL‐NP treatment (Figure 7h and Figure S14). Similarly, CXCL‐associated predicted communication showed reduced network connectivity in the APTL‐NP group (Figure 7i). These analyses nominate SPP1‐ and CXCL‐related signaling as candidate pathways potentially involved in the treatment‐associated remodeling of the post‐traumatic microenvironment.

Collectively, single‐cell profiling revealed that APTL‐NP treatment was associated with coordinated changes in myeloid, astrocytic, and vascular cellular states, accompanied by reduced inflammatory signatures and altered predicted intercellular communication. The inferred SPP1 and CXCL signaling axes should be interpreted as candidate mechanisms requiring direct experimental validation.

## 3 Discussion

Secondary neuroinflammation after traumatic brain injury is a major contributor to progressive tissue damage and long‐term neurological dysfunction, yet effective disease‐modifying therapies remain limited [34, 35, 36]. In this study, we identify infiltrating peripheral macrophages as the predominant TREM1‐expressing population in the TBI brain and establish TREM1 as an important amplifier of post‐TBI neuroinflammation. Building on this mechanistic insight, we integrated generative protein design, targeted receptor degradation, and BBB‐oriented nanodelivery to develop an Angiopep‐2‐functionalized, pH‐responsive TREM1‐LYTAC nanoplatform, APTL‐NP. This system enhanced delivery of the TREM1‐targeting degrader to the injured brain, reduced TREM1 abundance in vivo, attenuated inflammatory cytokine and chemokine responses, preserved neurovascular and neuronal integrity, and improved neurological recovery after TBI. Together, these findings provide a therapeutic framework for modulating secondary neuroinflammation through targeted regulation of a peripheral myeloid receptor.

A central finding of this study is that injury‐associated TREM1 in the TBI brain is largely linked to infiltrating monocyte‐derived macrophages rather than resident microglia. Previous studies have implicated TREM1 in inflammatory disorders of the central nervous system, but the cellular origin and functional contribution of TREM1‐expressing cells after TBI have remained incompletely defined [37, 38, 39]. By combining analysis of human TBI samples, public single‐cell transcriptomic datasets, Cx3cr1/Ccr2 reporter mice, and head‐protected bone marrow chimeras, we found that TREM1 expression was preferentially associated with peripheral myeloid‐lineage cells entering the injured brain. Functionally, Trem1 deficiency attenuated inflammatory activation without substantially reducing overall myeloid infiltration, suggesting that TREM1 primarily regulates the activation state of infiltrating myeloid cells rather than simply controlling their recruitment. The accompanying reductions in cytokine production, neuronal apoptosis, BBB disruption, edema, and neurological deficits further support TREM1 as a pathogenic amplifier of secondary brain injury. These results provide a rationale for therapeutically targeting TREM1‐positive peripheral myeloid cells in TBI.

Translating the protective phenotype of Trem1 deficiency into a pharmacological intervention remains challenging. Conventional TREM1 antagonists such as LP17 act through occupancy‐based receptor or ligand blockade and may require sustained exposure to maintain inhibitory activity during the rapidly evolving inflammatory response. In contrast, LYTAC‐based strategies offer a conceptually distinct approach by promoting internalization and lysosomal trafficking of extracellular or membrane‐associated proteins. In this study, we used RFdiffusion‐guided de novo binder design followed by computational and experimental screening to identify Pep457 as a TREM1‐binding peptide. Conjugation of Pep457 to mannose‐6‐phosphate generated a TREM1‐targeting LYTAC that reduced cellular TREM1 protein abundance through a predominantly lysosome‐dependent mechanism in vitro. This workflow illustrates how AI‐guided binder discovery can be combined with targeted protein degradation to create receptor‐directed degraders for inflammatory targets that have been difficult to modulate with conventional antagonists.

A second barrier to CNS application of macromolecular degraders is inefficient delivery across the BBB and limited payload release within diseased tissue. Stimuli‐responsive nanocarriers have been widely used to improve spatiotemporal control of drug release and reduce premature payload leakage under physiological conditions [40, 41]. APTL‐NP was designed to address these constraints by combining Angiopep‐2‐mediated BBB‐oriented delivery with pH‐responsive release in the acidic TBI lesion microenvironment. In vitro BBB‐model experiments showed increased delivery of APTL‐NP‐associated fluorescence to macrophages in the lower chamber without overt disruption of endothelial barrier integrity, as indicated by stable TEER values. In vivo, APTL‐NP produced stronger and more persistent fluorescence signals in the injured brain than the free binder and preferentially accumulated in IBA1^+^/CD11b^+^ myeloid‐lineage cells.

Importantly, the enhanced brain accumulation of APTL‐NP likely reflects not only Angiopep‐2‐mediated BBB targeting but also the pharmacokinetic advantages conferred by nanoformulation. Free TREM1‐LYTAC exhibited rapid systemic elimination, with an apparent plasma half‐life of approximately 1.13 h, whereas incorporation into APTL‐NP prolonged the apparent half‐life to approximately 8.38 h. APTL‐NP was constructed on a G4 PAMAM dendrimer scaffold, further modified by PEGylation, and exhibited a hydrodynamic diameter of approximately 180 nm. These physicochemical features are expected to substantially alter the systemic disposition of the peptide‐based degrader. Compared with free TREM1‐LYTAC, conjugation to the PAMAM‐based nanocarrier increases the effective hydrodynamic size of the payload, which is expected to reduce rapid renal filtration and may additionally provide partial steric protection against proteolytic degradation, consistent with experimental observations in other peptide–polymer and peptide–nanostructure systems [42]. Moreover, PEGylation can modulate nanoparticle–protein interactions and reduce rapid systemic clearance, thereby further contributing to prolonged blood circulation [43]. Thus, the extended systemic exposure of APTL‐NP is likely an intrinsic consequence of the nanocarrier formulation and may itself contribute to the increased accumulation observed in the TBI brain. Collectively, these findings suggest that the increased brain accumulation of APTL‐NP results, at least in part, from the prolonged systemic exposure conferred by nanoformulation, in addition to BBB‐targeting mechanisms, ultimately facilitating delivery to inflammatory myeloid cells within the TBI brain.

Single‐cell transcriptomic profiling further provided a systems‐level view of how APTL‐NP treatment reshapes the post‐traumatic neuroimmune microenvironment. Because this discovery dataset was generated from pooled samples, changes in cell‐state abundance and CellChat‐inferred communication should be interpreted as treatment‐associated descriptive patterns rather than definitive animal‐level statistical conclusions. Within this framework, APTL‐NP treatment was associated with a modest reduction in Apoe/Spp1‐associated DAM‐like myeloid cells, redistribution of astrocyte states involving homeostatic‐like, reactive, neuroprotective‐associated, and transitional populations, and relative preservation of BBB‐supportive endothelial programs. Rather than indicating a simple restoration of baseline astrocyte homeostasis, these findings suggest remodeling of the injury‐associated astrocytic response. Cell–cell communication analysis further indicated attenuation of SPP1‐ and CXCL‐associated signaling networks, nominating these pathways as potential mediators of the broader multicellular effects observed after TREM1 targeting. These transcriptomic results are consistent with the histological and functional evidence of reduced inflammatory activation and improved neurovascular integrity, while also highlighting candidate intercellular programs for future mechanistic validation.

Several limitations should be considered. First, although APTL‐NP reduced TREM1 abundance in vivo and our in vitro data support lysosome‐dependent degradation, additional studies using sorted myeloid populations will be needed to directly confirm lysosomal trafficking and degradation mechanisms in vivo. Second, the translational relevance of Pep457 requires further validation using human TREM1 protein and primary human myeloid cells, as the current human TREM1–Pep457 interaction is supported primarily by structural prediction. An additional structural consideration concerns the higher‐order organization of the PAMAM‐based nanoplatform. The approximately 180–191 nm size measured by DLS and TEM is substantially larger than the molecular dimensions expected for an individual G4 PAMAM dendrimer, suggesting that the final formulation comprises assemblies of multiple modified PAMAM units. Such higher‐order organization may arise from changes in hydrophilic–hydrophobic balance, surface charge, and multivalent intermolecular interactions introduced during functionalization. However, because TPA is a bifunctional aldehyde and the PAMAM–TPA intermediate undergoes reductive stabilization, a contribution from limited intermolecular covalent cross‐linking cannot be completely excluded. The current TEM, DLS, and ^1^H NMR analyses do not allow noncovalent supramolecular assembly to be quantitatively distinguished from potential covalent cross‐linking. Nevertheless, the final APTL‐NP population exhibited stable hydrodynamic size and polydispersity in both PBS and serum‐containing medium under the tested conditions. Further orthogonal physicochemical characterization will be required to define the molecular organization and degree of intermolecular cross‐linking within these assembled nanoparticles. Besides, longer‐term safety studies will be important to assess the consequences of repeated or sustained TREM1 suppression, particularly given the role of TREM1 in host defense. Future studies including female mice will be needed to evaluate potential sex‐dependent therapeutic effects.

## 4 Conclusions

This study identifies infiltrating peripheral macrophages as the predominant source of TREM1 in the injured brain and establishes TREM1 as a key amplifier of secondary neuroinflammation after TBI. By integrating AI‐guided peptide design, LYTAC‐mediated receptor degradation, and pH‐responsive nanodelivery, we developed APTL‐NP, a brain‐penetrating nanoplatform that reduces TREM1 abundance in vivo, attenuates neuroinflammation, preserves neurovascular integrity, and improves functional recovery. Single‐cell profiling reveals treatment‐associated remodeling of myeloid, astrocytic, and vascular states. Together, these findings establish targeted TREM1 reduction as a promising therapeutic strategy for TBI and offer a modular approach adaptable to other neuroinflammatory disorders involving peripheral immune infiltration.

## 5 Materials and Methods

### 5.1 Clinical Patient Samples Collection

5 mL of venous blood was collected from patients at 1,3,7, and 14 days post‐TBI. EDTA plasma was separated by centrifugation at 1000 × g for 10 min at 4°C and stored at −80°C for further analysis, while blood cells were used for flow cytometry (FC) analysis. Traumatic brain injury tissue samples were collected during emergency surgery for immunofluorescence (IF) analysis. All tissue samples were collected from injury sites during emergency neurosurgical procedures. In compliance with ethical guidelines, control brain tissues were obtained from non‐traumatized regions of the same patients' brains that required surgical resection for therapeutic decompression. The study was conducted in accordance with the Declaration of Helsinki and was approved by the Medical Ethics Committee of Tongji Hospital (approval no. TJH IRB 20230839). Written informed consent for the use of human tissue samples was obtained from all participants. Detailed clinical characteristics of the patient population are shown in Table S1.

### 5.2 Animals

All animal experiments were conducted in accordance with the ethical guidelines of the Experimental Animal Ethics Committee of Huazhong University of Science and Technology and were approved under protocol no. TJH 202201049. C57BL/6J mice were obtained from HuBei Biont Biological Technology Co., Ltd. Ccr2RFP/Cx3cr1GFP mice and EGFP C57BL/6J mice were purchased from Cyagen Biosciences Inc. Trem1 knockout mice were obtained from Shanghai Model Organisms Center, Inc. Genotyping was performed according to the manufacturers’ instructions. Mice were housed in the Experimental Animal Center of Tongji Scientific Building, Tongji Hospital, under controlled conditions of 22 ± 3°C, 60% relative humidity, and a 12‐h light/dark cycle, with free access to standard rodent chow and water.

Eight‐week‐old male mice weighing approximately 20 g were used for experimental procedures. Female mice were used only for breeding and were not included in the efficacy experiments to reduce variability associated with sex‐dependent hormonal cycles. Mice were randomly numbered and assigned to experimental groups. Investigators performing the TBI surgery and administering treatments were blinded to group allocation and treatment information.

### 5.3 TBI Model

The severe traumatic brain injury (TBI) model was performed according to established experimental protocols [44, 45]. Briefly, mice were anesthetized with 5% isoflurane in oxygen (0.5 L/min) for induction, followed by maintenance with 2% isoflurane. The mice were then positioned in a stereotaxic frame. After disinfecting the skin with alcohol, a sagittal incision was made along the midline. The scalp and fascia were retracted, and a 3 mm‐diameter craniotomy was performed over the left parietal cortex, located 2.5 mm posterior to the bregma and 2.5 mm lateral to the midline suture, using a portable drill. The skull was carefully removed, avoiding damage to the dura mater. To induce a unilateral, severe CCI (YHCI99; Wuhan Yihong Technology Co., Ltd.), a 3‐mm flat impactor tip was used to impact the exposed dura (impact parameters: velocity: 5 m/s, depth: 3 mm, dwell time: 500 ms; zero‐point demarcation at the skull). After the impact, the injured mice were sutured without replacing the skull cap over the craniectomy site and placed on a warming pad, and sustained‐release buprenorphine was administered subcutaneously at a dose of 0.5 mg/kg as a single dose immediately after surgery, providing analgesia for approximately 72 h. Sham‐operated animals underwent the same procedure except for the CCI.

### 5.4 Measurement of Lesional pH After Traumatic Brain Injury

At designated time points after traumatic brain injury (TBI), mice were anesthetized and the skin overlying the lesion was incised to expose the injury site; the cortical surface was continuously superfused with artificial cerebrospinal fluid (ACSF) to maintain moisture, and a portable handheld pH meter equipped with a planar glass pH electrode was gently applied perpendicularly to the center of the lesion to measure extracellular pH. The probe was allowed to equilibrate until a stable reading was obtained, and the measurement was repeated 2–3 times and averaged for analysis; the pH meter was calibrated with standard buffers (pH 4.00, 7.00, and 9.00) at the experimental temperature prior to each session, and care was taken to avoid pressure artifacts or bleeding during probe contact

### 5.5 De Novo Design and Screening of TREM1‐Targeting Peptides via Artificial Intelligence

To engineer *de novo* binders targeting the Ig‐like V‐type domain of TREM1, we employed the RFdiffusion model to generate 1000 candidate peptide backbones with lengths ranging from 20 to 50 amino acids via a reverse diffusion process initialized from Gaussian noise. These candidates were subjected to a rigorous structural validation pipeline integrating AlphaFold 3 (AlphaFold Server, accessed in November 2025), ZDOCK (version 3.0.2), and HDOCK (HDOCK server, accessed in November 2025), where a stringent filtration threshold of predicted aligned error (pae_interaction) < 10 was applied to ensure high‐confidence interfacial interactions. The top 10 candidates emerging from this structural screening were subsequently refined using Rosetta‐based energy analysis to optimize thermodynamic stability and binding affinity, culminating in the identification of the top 3 peptide binders for experimental characterization.

### 5.6 Synthesis of TREM1‐Targeting Lysosomal Targeting Chimeras (TREM1‐LYTAC)

The TREM1‐targeting chimera was synthesized by coupling M6P moieties to the peptide backbone via on‐resin CuAAC [46]. Briefly, the peptide sequence (MVVEKTVKPVDPELAAAWDAERRAA) was elongated on Rink amide resin (0.54 mmol/g) using standard Fmoc chemistry, followed by the incorporation of a PEG3 linker and three Pra‐βAla motifs to introduce reactive alkyne handles. Conjugation was initiated by incubating the resin‐bound peptide with M6P‐azide (2.0 equiv.) in the presence of CuSO_4_, sodium ascorbate, and TBTA in aqueous DMF. After 36 h of agitation at room temperature, the resin was washed to remove excess reagents. The final construct was cleaved from the solid support using a TFA‐based solution (95% TFA, 2.5% triethylsilane, 2.5% H2O), precipitated in ether, and purified to homogeneity (>95% purity) using semi‐preparative RP‐HPLC.

### 5.7 Synthesis of APTL‐NP

The multifunctional TREM1‐LYTAC nanoparticles were synthesized via a stepwise conjugation strategy based on a Generation 4 (G4) PAMAM dendrimer scaffold. Initially, terephthalaldehyde (TPA) was coupled to the PAMAM surface amines via Schiff base formation under mild conditions, followed by the specific attachment of the TREM1‐LYTAC to the aldehyde terminals of the TPA linker. Subsequently, Angiopep‐2 was activated using EDC/NHS chemistry in MES buffer and covalently conjugated to the remaining PAMAM surface amines via stable amide bonds. To enhance biocompatibility and targeting capability, the construct was further surface‐modified with PS‐PEG‐NHS. All intermediates and the final product were purified by dialysis (MWCO 3.5 kDa) at 4°C to remove unreacted small molecules and lyophilized for storage. The physicochemical properties of the synthesized nanoparticles were characterized by ^1^H‐NMR spectroscopy for structural validation, while their morphology and hydrodynamic size were assessed using transmission electron microscopy (TEM) and dynamic light scattering (DLS), respectively (The step‐by‐step protocol is available in Supplementary Information S1).

### 5.8 TRIC

Binding affinities of the synthesized peptides (Pep 15, Pep 17, and Pep 457) toward the target protein were characterized using a Dianthus NT.23 instrument (NanoTemper Technologies, Munich, Germany). The target protein was fluorescently labeled via amine‐reactive chemistry using the Monolith Protein Labeling Kit RED‐NHS second Gen (cat#MO‐L011, NanoTemper Technologies, Munich, Germany). For the binding assay, a fixed concentration of the labeled protein (10 nM) was mixed with a 16‐step serial dilution of the respective peptides in assay buffer (PBS supplemented with 0.05% Tween‐20). The samples were incubated at room temperature to reach thermodynamic equilibrium and subsequently loaded into a 384‐well plate. TRIC measurements were performed with the excitation power optimized to maintain initial fluorescence counts between 2000 and 15 000. Data analysis was conducted using the Dianthus analysis software, where the normalized fluorescence intensity (Fnorm) was plotted against the logarithm of the ligand concentration (fnorm(%) = 100 × (Fsample − Fmin)/(Fmax−Fmin))

### 5.9 Surface Plasmon Resonance (SPR) Kinetic Analysis

Real‐time binding kinetics between the analyte and the immobilized target protein were characterized using a Biacore T200 system (Cytiva, Uppsala, Sweden). The target protein was covalently coupled to a Series S Sensor Chip CM5 via standard amine coupling chemistry, achieving a final immobilization level of approximately 2000 resonance units (RU). For kinetic measurements, the analyte was diluted in the running buffer to generate a five‐point concentration series. The samples were injected over the reference and active flow cells at a flow rate of 30 µL/min at 25°C. The association phase was monitored for 120 s, followed by a dissociation phase of 180 s with running buffer. The resulting sensorgrams were double‐referenced by subtracting the signals from the control flow cell and buffer blanks. Kinetic rate constants and the equilibrium dissociation constant were derived by globally fitting the data to a 1:1 Langmuir binding model using the Biacore Evaluation Software.

### 5.10 Enzyme‐Linked Immunosorbent Assay (ELISA)

The binding affinity of the Fc‐fusion peptide variants (Pep 15, Pep 17, and Pep 457) toward the TREM1 receptor was assessed using a direct binding ELISA. High‐binding 96‐well microplates were coated with the TREM1 protein (1 µg/mL in carbonate‐bicarbonate buffer, pH 9.6) overnight at 4°C. Following surface passivation with 2% bovine serum albumin in PBST to prevent non‐specific adsorption, the plates were incubated with serial dilutions of the candidate Fc‐binders for 1 h at 37°C. Bound ligands were detected using a horseradish peroxidase (HRP)‐conjugated goat anti‐mouse IgG secondary antibody. The colorimetric reaction was developed with 3,3′,5,5′‐tetramethylbenzidine (TMB) substrate and terminated by the addition of 2 M H2SO4. Optical density (OD) was measured at 450 nm using a microplate reader, and binding curves were generated by plotting absorbance values against ligand concentration.

Brain tissue was sonicated on ice in RIPA lysis buffer (Cat# G2002; Wuhan Servicebio Technology Co., Ltd., China) prepared with PMSF and phosphoprotein inhibitors (RIPA: PMSF: phosphoproteins inhibitor A: B = 100:1:1:1). The homogenates were centrifuged at 14 000 × g for 10 min at 4°C, and the supernatants were collected and stored at −20°C. The total protein concentration was determined using the BCA method. ELISA kits were used for the quantification of the following mouse cytokines and proteins: IL‐1β, TNFα, IL‐10, IL‐6, MCP‐1, MMP3, TGFβ, and TREM1. For human TREM1, the kit used was Cat# ab270884 (Abcam, Cambridge, UK). All ELISAs were performed in duplicate according to the manufacturer's instructions, and concentrations were normalized to the total protein content.

### 5.11 Microscale Thermophoresis (MST) Binding Assay

The equilibrium dissociation constants for the interactions between the TREM1 targeting ligands (TREM1‐binder and TREM1‐binder‐M6P3) and the TREM1 protein were quantified using a Monolith NT.115 instrument (NanoTemper Technologies, Munich, Germany). The target protein was fluorescently labeled via surface lysine residues using the Monolith Protein Labeling Kit RED‐NHS second Gen (cat#MO‐L011). For the binding measurements, a fixed concentration of the labeled rhTREM1 (final concentration: 20 nM) was incubated with a 16‐point serial dilution of the respective ligand in assay buffer (PBS supplemented with 0.05% Tween‐20) at room temperature. The samples were loaded into standard treated capillaries, and thermophoretic movement was monitored with the excitation power set to 40% and MST power set to medium. Data analysis was performed using the MO. Affinity Analysis software, where the Fnorm or fraction bound was plotted against ligand concentration.

### 5.12 Apparent Estimation of Drug Plasma Half‐life

To estimate the apparent plasma half‐life of Cy5.5‐labeled free LYTAC and APTL‐NP, plasma fluorescence intensity was measured using a microplate reader at the indicated time points after intravenous injection. Blank plasma from untreated mice was used for background subtraction. The corrected fluorescence intensity at each time point was normalized to the value at 5 min post‐injection and plotted as a function of time. The decay curves were fitted using a one‐phase exponential decay model.

### 5.13 pH‐Dependent Release of Bioactive Peptide

The pH‐responsive release of TREM1‐LYTAC from APTL‐NP was evaluated in PBS adjusted to pH 7.5, 7.0, or 6.5. APTL‐NP was suspended in each buffer at a concentration of 1 mg/mL and incubated at 37°C under gentle agitation. At predetermined time points (2, 6, 12, and 24 h), aliquots were collected and immediately subjected to ultrafiltration using a 100 kDa MWCO centrifugal filter, which was pre‐validated to retain APTL‐NP while allowing free TREM1‐LYTAC to pass through into the filtrate. The filtrates containing released free TREM1‐LYTAC were collected and neutralized to pH 7.4 before analysis.

The concentration of released bioactive TREM1‐LYTAC was quantified using a TREM1‐binding ELISA. Briefly, 96‐well plates were coated with recombinant TREM1 extracellular domain protein and blocked with 5% BSA. Filtrate samples and serial dilutions of free TREM1‐LYTAC standards were added to the wells. The amount of TREM1‐LYTAC in each sample was determined from the standard curve generated using free TREM1‐LYTAC standards and expressed as the cumulative percentage of the total TREM1‐LYTAC initially loaded into APTL‐NP.

To exclude potential interference from pH exposure or ultrafiltration, free TREM1‐LYTAC incubated under identical pH conditions and subjected to the same ultrafiltration procedure was analyzed in parallel.

### 5.14 BBB Crossing Efficiency In Vitro

An in vitro blood–brain barrier (BBB) model was established using Transwell inserts to evaluate the BBB penetration efficiency of the peptide or peptide‐loaded nanocarrier formulations. Briefly, bEnd.3 brain endothelial cells (Cat #CRL‐2299, ATCC) were seeded onto the upper side of the Transwell membrane inserts with a pore size of 0.4 µm and cultured for 3 days until a tight and confluent endothelial monolayer was formed. RAW264.7 cells (Cat #TIB‐71, ATCC) were seeded in the basolateral lower chamber and cultured for 24 h before the transport assay. FITC‐labeled peptides and peptide‐containing nanocarriers were then added to the apical upper chamber and incubated for 12 h at 37°C in a humidified atmosphere containing 5% CO_2_. After incubation, the lower chamber medium and RAW264.7 cells were collected to evaluate the translocation of peptides across the in vitro BBB model.

The integrity of the in vitro BBB model was evaluated by trans‐endothelial electrical resistance (TEER) before and after nanoparticle treatment. TEER was measured using an epithelial voltohmmeter equipped with STX chopstick electrodes. Only endothelial monolayers with baseline TEER values ≥40 Ω cm^2^ and within the stable pre‐experimental range of 40–70 Ω cm^2^ were included in the BBB transcytosis assay. After treatment, TEER was monitored at 0, 6, 12, and 24 h to determine whether nanoparticle transport altered barrier integrity.

### 5.15 Fluorescent Labeling of Peptide

For site‐specific fluorescent labeling, a cysteine residue separated from the functional peptide sequence by an aminohexanoic acid (Ahx) spacer was introduced at the N‐terminus of the TREM1‐binding peptide (Cys‐Ahx‐MVVEKTVKPVDPELAAAWDAERRAA). The peptide was dissolved in PBS (pH 7.0–7.2) and incubated with TCEP at a peptide‐to‐TCEP molar ratio of 1:5 for 30 min at room temperature to reduce the cysteine thiol. After removal of excess TCEP, either fluorescein‐5‐maleimide or Cy5.5‐maleimide was added at a fluorophore‐to‐peptide molar ratio of 1.1–1.2:1. The reaction was allowed to proceed for 2 h at room temperature in the dark. The labeled peptides were purified by C18 reverse‐phase HPLC and characterized by analytical HPLC and LC–MS. Only preparations with ≥95% purity and predominantly 1:1 fluorophore‐to‐peptide conjugation were used for subsequent experiments. Fluorescein‐labeled formulations were used for in vitro fluorescence and BBB‐transport experiments, whereas Cy5.5‐labeled formulations were used for in vivo fluorescence imaging, biodistribution, and tissue localization analyses. For comparative biodistribution experiments, the same batch of site‐specifically Cy5.5‐labeled peptide was used to prepare the corresponding free and nanoparticle formulations, and all groups were normalized to an equivalent amount of Cy5.5‐labeled peptide.

### 5.16 Cell Treatment and Degradation Assays

Bone marrow‐derived macrophages (BMDMs) were differentiated and subsequently primed with lipopolysaccharide (LPS, 500 ng/mL) for 24 h to induce robust TREM1 expression. To evaluate dose‐dependent degradation efficacy, the activated BMDMs were incubated with serial dilutions (0.2–50 µM) of TREM1‐LYTAC or the Scrambled‐LYTAC control for a defined duration. Total TREM1 protein levels in the cell lysates were quantified using a commercial ELISA kit, with concentrations normalized to total protein content. For the assessment of surface receptor downregulation, cells treated with Vehicle, LPT, or LYTAC were harvested and stained with a fluorophore‐conjugated anti‐TREM1 antibody. The surface abundance of TREM1 was analyzed via flow cytometry, where the median fluorescence intensity (MFI) shifts were recorded to quantify the extent of receptor depletion.

### 5.17 Intracellular Trafficking and Colocalization Analysis

To elucidate the lysosomal routing mechanism, LPS‐stimulated BMDMs were seeded on confocal dishes and treated with fluorescently labeled TREM1‐LYTAC. Following incubation, lysosomes were labeled using LysoTracker Red (#C1046, Beyotime, China), and nuclei were counterstained with DAPI. Live‐cell or fixed‐cell imaging was performed using a confocal laser scanning microscope. The intracellular spatial distribution of the LYTAC complex was analyzed using ImageJ software, where the degree of lysosomal sequestration was determined by evaluating the fluorescence overlap.

### 5.18 In Vivo Biodistribution and Cellular Tropism Analysis

To evaluate brain‐targeting efficiency, TBI mice received intravenous injections of the indicated Cy5.5‐labeled formulations. The same site‐specifically Cy5.5‐labeled peptide preparation was used to generate the corresponding free and nanoparticle formulations, and all formulations were administered at an equivalent dose of Cy5.5‐labeled peptide to ensure comparable fluorescent input among groups. Whole‐body fluorescence imaging was performed at the indicated time points using an IVIS imaging system. Following the final imaging time point, mice were euthanized, and the heart, liver, spleen, lung, kidney, and brain were harvested for ex vivo fluorescence imaging. Fluorescence signals were quantified using identical acquisition settings and predefined regions of interest across experimental groups.

### 5.19 Erythrocyte Compatibility Test

Fresh mouse blood was collected and mixed with an anticoagulant, followed by centrifugation at 3500 rpm for 30 min. The supernatant was discarded, and the pellet was washed three times with phosphate‐buffered saline (PBS), The obtained erythrocytes were diluted 10‐fold with PBS to prepare an erythrocyte suspension. The 200 µL of the diluted erythrocyte suspension was respectively added to 1 mL of Triton X‐100 (positive control), PBS (negative control), and APTL‐NP dispersion solutions at various concentrations for the experimental group. Samples were incubated at 37°C for 5 h, then centrifuged at 3000 rpm (when photos were taken). 100 µL of the supernatant was transferred to a 96‐well plate. The absorbance (OD value) of the supernatant was measured at 541 nm using a microplate reader. The hemolysis rate was calculated as: Hemolysis Rate = (ODexperimental—ODnegative) / (ODpositive—ODnegative) *100%

### 5.20 Sample Preparation—Brain

After perfusion with cold PBS, the brains were entirely removed from the skulls for immunofluorescence (IF) analysis. They were fixed with 4% paraformaldehyde (PFA) overnight and then cryoprotected by immersion in a 30% sucrose solution for 48 h. The brains were embedded in Tissue‐Tek O.C.T. Compound (SAKURA, 4583) and sectioned into 10 µm thick coronal slices using a freezing microtome (Leica, Wetzlar, Germany). For biochemical analysis, brains were immediately snap‐frozen in liquid nitrogen and stored at −80°C for subsequent protein and RNA extraction.

For FC, brains were removed and bisected along the midline. One hemisphere (either lesion lateral or sham) was taken for FC analysis, excluding the olfactory bulb and cerebellum. The tissue was placed in a tube grinder (C‐Tube, Cat# 130‐107‐677, Miltenyi) and processed using the gentleMACS Octo Dissociator with Heaters. A MACS Smart Strainer (70 µm) was used to obtain a single‐cell suspension, followed by centrifugation at 400 × g for 20 min at 4°C to pellet the cells. The cells were resuspended in 30% Percoll solution (Cat# 17089102, Cytiva, St. Louis, USA) and centrifuged at 950 × g (acceleration 4, deceleration 0) for 20 min to remove myelin debris. The resulting cell pellet was resuspended in 200 µL of FACS buffer (PBS with 2% BSA and 5 mM EDTA), and cell counts were performed using a Neubauer chamber.

### 5.21 Sample Preparation—Blood

Whole blood from mice was collected into EDTA‐coated tubes (BD Microtainer K2E tubes; BD Biosciences) via the inferior vena cava. After red blood cell lysis and centrifugation at 2000 rpm for 10 min at 4°C, single cells were collected for further processing.

### 5.22 Flow Cytometry

Single‐cell suspensions were prepared and stained in FACS buffer for 20 min on ice with the indicated antibodies. For macrophage TREM1 expression analysis, monoclonal antibodies including anti‐CD11b (M1/70), CD45, and TREM1 were used. For peripheral‐derived mononuclear macrophage analysis, monoclonal antibodies including anti‐CD11b (M1/70), CD45, TREM1, Ly6C, and CCR2 were utilized. All cells were stained on ice for 30–50 min. Data were acquired using a Beckman CytoFLEX flow cytometer and analyzed with FlowJo software (TreeStar). To exclude dead cells and cell aggregates, the Zombie Aqua Fixable Viability Kit (Cat# 423101, BioLegend, California, USA) and FSC‐H/FSC‐A characteristics were used.

### 5.23 EdU Staining Experiment

Upon reaching an appropriate confluency after drug administration 24 h, BMEC (Cat #C57‐6023, Cell Biologics), HT22 (Cat# SCC129, Sigma‐Aldrich), and NHA (Cat #CC‐2565, Lonza) were fixed with 4% paraformaldehyde at room temperature for 15 min. Following fixation, cells were blocked with a solution containing 5% bovine serum albumin (BSA) and 0.2% Triton X‐100 for 2 h. EdU staining was then performed according to the manufacturer's instructions (Cat# KTA2031, Abbkine, China). Finally, images were captured under a light microscope, and the positive cell rate was quantified using ImageJ software.

### 5.24 Apoptosis Assay

Twenty‐four hours after drug administration, once the cells reached an appropriate confluency, they were detached by trypsinization. The cell pellet was collected by centrifugation, washed three times with PBS, and then subjected to apoptosis staining according to the kit instructions (Cat# KTA0005, Abbkine, China). Apoptotic cells were analyzed by flow cytometry.

### 5.25 Bone Marrow Transplantation

Bone marrow transplantation was performed as previously described with minor modifications [47, 48]. Recipient mice were subjected to head‐protected irradiation with a total dose of 9 Gy delivered in two fractions. Approximately 2 h after irradiation, mice were anesthetized and intravenously injected via the tail vein with approximately 1 × 10^6^ bone marrow cells isolated from donor mice. To improve post‐irradiation survival and transplantation efficiency, approximately 25% syngeneic radioprotective support cells were co‐transplanted when indicated. For GFP bone marrow chimera experiments, irradiated WT recipients received bone marrow cells from EGFP‐transgenic C57BL/6J donor mice. For Trem1 bone marrow chimera experiments, irradiated WT recipients were reconstituted with bone marrow cells from either WT or *Trem1* ^−/−^ donor mice. *Trem1* ^−/−^ mice subjected to TBI were included as a genetic‐deficiency reference group. Chimerism was assessed 2 months after transplantation, and only mice with successful hematopoietic reconstitution were used for subsequent CCI experiments.

### 5.26 Immunofluorescence Staining

Sections of brain and spleen tissue were blocked with 5% normal donkey serum for 2 h at room temperature. The sections were then incubated overnight with the primary antibody. On the following day, sections were washed with PBS and incubated with the secondary antibodies: anti‐rabbit IgG (H + L), F(ab’)2 fragment conjugated to Alexa Fluor 594, and anti‐mouse IgG (H + L), F(ab’)2 fragment conjugated to Alexa Fluor 488 for 1 h at room temperature. SuperKine Enhanced Antifade Mounting Medium with DAPI was applied before sealing with a coverslip.

### 5.27 TUNEL Assay

To detect neuronal apoptosis in the brain following TBI, a TUNEL assay was performed according to the manufacturer's instructions. Fluorescence microscopy was performed to image three randomly selected fields surrounding the lesion area in each TBI brain section. Neuronal apoptosis was assessed by determining the percentage of TUNEL‐positive neurons among total neurons using ImageJ analysis.

### 5.28 Western Blot

Western blotting was performed as previously described. Briefly, total protein from BMDM cells or tissue samples was extracted using RIPA lysis buffer (Cat# G2002; Wuhan Servicebio Technology Co., Ltd., China) with PMSF and phosphoprotein inhibitors (RIPA: PMSF: phosphoproteins inhibitor A: B = 100:1:1:1) on ice. Protein concentrations were determined using the BCA method, and samples were denatured by boiling for 10 min. Equal amounts (20 µg) of total protein were separated by SDS‐PAGE on 10% gels and transferred onto Immobilon‐P PVDF membranes (Cat# IPVH00010; Millipore Sigma, USA). The membranes were then blocked with NcmBlot blocking buffer (Cat# P30500; NCM Biotech, China) for 15 min and incubated with primary antibodies overnight. The following day, secondary antibodies (HRP‐Goat anti‐rabbit, HRP‐Goat anti‐mouse, 1:50000; Searcare) were incubated at room temperature for 1 h. BeyoECL reagent (Cat# P0018S; Beyotime Institute of Biotechnology, China) was used for protein visualization, and the signals were detected using the GeneGnome XRQ Chemiluminescence Imaging System (GeneGnome XRQ; Syngene). Band intensity was quantified using ImageJ 1.8.0 software.

### 5.29 Single‐Cell Suspension Preparation and scRNA‐seq Library Construction

Ipsilateral cortical tissues were collected at 3 days after TBI from Vehicle‐ and APTL‐NP‐treated mice. For each treatment condition, ipsilateral cortices from three mice were pooled to generate one composite sample for single‐cell RNA sequencing. Thus, the scRNA‐seq dataset comprised one pooled library per treatment group and was used for exploratory characterization of treatment‐associated cellular and transcriptional patterns.

Harvested tissues were washed in ice‐cold PBS (Hyclone, Cat. No. SH30256.01) and dissociated using the SeekMate Tissue Dissociation Reagent Kit A Pro (SeekGene, Cat. No. *K01801301*) or SeekMate Tissue Dissociation Kit C (SeekGene, Cat. No. K01501) following the manufacturer's instructions. Depending on homogenate viscosity, samples were optionally treated with DNase I (Sigma, Cat. No. 9003‐98‐9). Following erythrocyte lysis (Solarbio, Cat. No. R1010) and optional removal of debris and dead cells (Miltenyi, Cat. No. 130‐109‐398/130‐090‐101), cell viability and concentration were assessed using a Fluorescence Cell Analyzer (Countstar Rigel, S2) or SeekMate Tinitan Fluorescence Cell Counter (SeekGene, M002C) with AO/PI staining. Fresh cells were washed twice with RPMI1640 (Gibco, Cat. No. 11875119) and resuspended at a density of 1 × 10^6^ cells/mL in RPMI1640 supplemented with 2% FBS (Gibco, Cat. No. 10100147C). Single‐cell RNA‐seq libraries were generated using the SeekOne Digital Droplet Single Cell 3' library preparation kit (SeekGene, Cat. No. K00202). Briefly, cells were mixed with reverse transcription reagents and co‐encapsulated with Barcoded Hydrogel Beads (BHBs) into a SeekOne Chip S3. Reverse transcription was performed at 42°C for 90 min and inactivated at 85°C for 5 min. The cDNA was purified, PCR‐amplified, fragmented, end‐repaired, and ligated to sequencing adaptors. Indexed PCR was performed to amplify the 3' polyA region containing Cell Barcodes and Unique Molecular Indices (UMIs). Final libraries were purified using VAHTS DNA Clean Beads (Vazyme, Cat. No. N411‐01), quantified by Qubit (Thermo Fisher Scientific, Cat. No. Q33226), and characterized using a Bio‐Fragment Analyzer (Bioptic, Qsep400). The single‐cell RNA‐seq library construction and sequencing services were provided by SeekGene (Beijing, China).

### 5.30 Single‐Cell RNA Sequencing Analysis

Raw data were obtained from the Gene Expression Omnibus (GEO: GSE175430, GSE209552, GSE125976, GSE271769, GSE226207, GSE266610, and GSE180862) and SeekGene. The R package Seurat (v.4.2.2) was used for data processing and analysis. Filters were set to include only genes detected in at least three cells, and cells containing a minimum of 200 genes, while excluding cells with mitochondrial gene expression ≥5%, ≥4500 genes, and ≥10 000 UMIs. For dimensionality reduction, 15 dimensions were used for integration and downstream analysis, including clustering and visualization via t‐SNE. Clusters were identified using graph‐based clustering at various resolutions. The FindMarkers function in Seurat was used to determine marker genes for each cluster, which were then labeled by cell type and subtype. Dot plots and violin plots were generated with ggplot2, and GSEA was performed to identify significantly enriched pathways.

### 5.31 Magnetic Resonance Imaging (MRI)

Small animal MRI was performed using a Bruker 9.4 T magnet (Bruker, Biospec 94/40 USR, Germany) controlled by Paravision 6.0.1 software. The 2D coronal T2‐weighted (T2W) images were acquired using the RARE (Rapid Acquisition with Relaxation Enhancement) pulse sequence with the following parameters: Echo Time = 60 ms, Repetition Time = 3000 ms, Averages = 10, Repetitions = 1, Rare Factor = 8, Image Size = 256 × 256, Bandwidth = 50 000 Hz, Slice Thickness = 1.5 mm, Field of View (FOV) = 2 × 2 cm, Refocusing flip angle = 180°, and an estimated total scan time of 16 min and 8 s. The Edema volume = longest axis (cm) * longest axis perpendicular to longest axis (cm) * number of slices × slices thickness (cm).

### 5.32 Water Content Assessment

After sacrifice, the brain was removed, and the injured hemispheres were dissected and weighed to obtain the wet weight. After drying at 105°C for 24 h, the dry weight was recorded. The severity of brain edema was calculated using the formula:

$$\begin{array}{ccc} \mathrm{Brain}\;\mathrm{water}\;\mathrm{content}\;\left(\%\right) & = & \left(\mathrm{wet}\;\mathrm{weight}-\mathrm{dry}\;\mathrm{weight}\right)\;\\ & & /\;\mathrm{wet}\;\mathrm{weight}\;\times \;100\% \end{array}$$

### 5.33 Evans Blue

At 1d post‐TBI, 2% Evans blue dye was injected through the tail vein and allowed to circulate for 2 h. Following perfusion with cold PBS to remove intravascular Evans blue dye, the ipsilateral brain tissue was homogenized and centrifuged at 12 000 × g for 30 min at 4°C. The supernatant was mixed with an equal volume of a trichloroacetic acid and ethanol mixture and incubated overnight at 4°C. After another centrifugation, the supernatant was collected, and the optical density (OD) value was measured with a spectrophotometer at a wavelength of 610 nm.

### 5.34 Modified Neurological Severity Score (mNSS)

The modified Neurological Severity Score (mNSS) was recorded at 1, 3, and 7 days post‐TBI, assessing sensory, motor, balance, and reflex functions. Neurological function was graded on a scale of 0–18, with higher scores indicating greater impairment, and behavioral assessors only had access to mouse identification numbers, which adhered to blinding protocols

### 5.35 Rotarod Test

The Rotarod test was used to evaluate motor coordination, limb strength, and balance. In the fixed‐speed test, mice were placed on the Rota‐Rod Treadmill (ZH600‐B, Zhenghua Biological Instrument Equipment Co., Ltd., China) set at a rotation speed of 30 rpm for 5 min. In the accelerating test, mice were placed on the swivel bar fatigue tester, with the speed gradually increasing from 4 rpm (initial velocity of 5 rpm for the first 10 s) to 40 rpm over a period of 300 s. The experiment was repeated three times with at least 15‐min intervals, and the longest duration each mouse stayed on the bar was recorded as the evaluation value.

### 5.36 Open‐Field Test (OFT)

The apparatus consisted of a white opaque square arena (Shanghai Xinruan Information Technology Co., Ltd., Shanghai, China) with dimensions of 40 × 40 × 40 cm. The floor of the arena was virtually divided into nine equal squares (3 × 3 × 3 grid). The central square was defined as the “central zone,” while the remaining eight squares constituted the “peripheral zone.” Mice were acclimatized to the testing room for at least 30 min prior to the experiment. For testing, each mouse was gently placed in the center of the arena and allowed to explore freely for 5 min. The apparatus was cleaned with 70% ethanol between trials to eliminate olfactory cues. The movement of the mice was recorded by a video camera and analyzed using VisuTrack (Shanghai Xinruan Information Technology Co., Ltd.). Key parameters, including total distance traveled and time spent in the central zone, were calculated.

### 5.37 Key Resources

Key reagents and analytical resources used in this study are summarized in Table 1, including antibodies, peptides, commercial assay kits, deposited datasets, and analysis software. These resources were used for myeloid‐cell characterization, biochemical and histological assessment of TBI and treatment responses, and single‐cell transcriptomic and quantitative analyses. The table provides the corresponding sources, identifiers, dataset accession numbers, and software information to facilitate experimental reproducibility and data traceability.

**TABLE 1:** Key resources table.

REAGENT or RESOURCE	SOURCE	IDENTIFIER
**Antibodies**		
FITC anti‐human CD45	BioLegend	368508
PE anti‐human TREM1	BioLegend	314906
APC anti‐mouse/human CD11b	BioLegend	101211
APC/Cyanine7 anti‐mouse CD45	BioLegend	103116
PE/Cyanine7 anti‐mouse CD45.1	BioLegend	110730
PE anti‐mouse Ly‐6c	BioLegend	128007
PE anti‐mouse CCR2	BioLegend	150609
BV421 Anti‐Mouse TREM1	BD Pharmingen	747899
TREM1 Polyclonal antibody	Proteintech	11791‐1‐AP
Synaptophysin Polyclonal antibody	Proteintech	17785‐1‐AP
GAPDH Monoclonal antibody	Proteintech	60004‐1‐Ig
βIII‐Tubulin Rabbit mAb	ABclonal	A17913
Occludin Polyclonal Antibody	Thermo Fisher	404700
Anti‐TNF‐alpha Rabbit pAb	Servicebio	GB11188‐100
Anti‐PSD95	Abcam	Ab18258
HRP‐Goat anti‐rabbit	Proteintech	SA00001‐2
HRP‐Goat anti‐mouse	Proteintech	SA00001‐1
anti‐rabbit 594	CST	#8889
anti‐mouse 488	CST	#4408
**Chemicals, peptides, and recombinant proteins**		
Angiopep‐2	MCE	# HY‐P1351
LP17	Shanghai Science Peptide Biological Technology Company	N/A
**Critical commercial assays**		
IL‐1β ELISA kit	ABclonal	# RK00006
TNFα ELISA kit	ABclonal	# RK00027
IL‐10 ELISA kit	ABclonal	# RK00016
IL‐6 ELISA kit	Elabscience	#E‐HSEL‐M0003
MCP‐1 ELISA kit	Elabscience	# E‐UNEL‐M0077
MMP3 ELISA kit	Elabscience	# E‐EL‐M0626
TGFβ ELISA kit	Elabscience	# E‐UNEL‐M0099
C35 ELISA Kit	Elabscience	# E‐EL‐M0339
C3a ELISA Kit	Elabscience	# E‐EL‐M0337
TREM1 ELISA kit	Abcam	# ab270884
TREM1 ELISA kit	Keshun Science and Technology	# KS18245
**Deposited data**		
scRNA‐seq data for TBI cortex	GEO‐NCBI	GSE175430, GSE209552, GSE125976, GSE271769, GSE226207, GSE266610, GSE180862
scRNA‐seq data for TBI treatment cortex	GSA	subCRA065396
**Software and algorithms**		
GraphPad Prism 8	GraphPad	https://www.graphpad.com
R package Seurat (v.4.2.2)	Satija Lab	https://satijalab.org/seurat/
R (v4.2.1)	The R Foundation for Statistical Computing	https://www.r‐project.org/
DESeq2 (v1.26.0)	Bioconductor	https://bioconductor.org/packages/release/bioc/html/DESeq2.html
Imaris (v.10.4.2)	Oxford Instruments	https://imaris.oxinst.com/
ImageJ (v.1.54f)	National Institutes of Health	https://imagej.nih.gov/ij
Pymol (v.3.03)	Pymol	https://pymol.org/
FlowJo software (v.10.4.2)	BD Biosciences	https://www.flowjo.com/solutions/flowjo

### 5.38 Statistical Analysis

All studies were conducted using randomization and blinding procedures where applicable. Statistical analyses were performed using GraphPad Prism 8 (GraphPad Software, San Diego, CA, USA). Data preprocessing and assay‐specific normalization, where applicable, were performed as described in the corresponding Experimental subsections. Normality of data distribution was assessed using the Shapiro–Wilk test before selection of statistical tests. No data points were excluded on the basis of statistical outlier testing.

Data are presented as mean ± SD unless otherwise indicated. The sample size (n) and experimental unit for each analysis are specified in the corresponding figure legends and Source Data. For animal experiments, n represents the number of individual animals, whereas for in vitro experiments, n represents the number of independent experimental replicates unless otherwise specified. Technical replicates, where applicable, were averaged and were not treated as independent experimental units.

For normally distributed data, comparisons between two independent groups were performed using an unpaired two‐tailed Student's t‐test, whereas paired two‐tailed Student's t‐tests were used for matched observations. Comparisons among three or more groups involving a single experimental factor were performed using one‐way analysis of variance, and experiments involving two experimental factors or longitudinal measurements were analyzed using two‐way ANOVA. Tukey's multiple‐comparison test was used for post‐hoc pairwise comparisons following ANOVA. For data that did not meet the assumption of normality, comparisons between two independent groups were performed using a two‐tailed Mann–Whitney U test. The assumptions underlying the selected statistical tests were evaluated before analysis, including assessment of data distribution using the Shapiro–Wilk test.

All statistical tests were two‐sided unless otherwise indicated, and the significance level was set at α = 0.05. Exact *p* values are provided in the Source Data where applicable. Statistical significance is indicated as follows: ns, not significant; *p* < 0.05, *p* < 0.01, *p* < 0.001, and *p* < 0.0001. The statistical test, sample size (n), data presentation, and definitions of significance symbols are additionally specified in the corresponding figure legends.

### 5.39 Ethics Approval and Consent to Participate

All experimental animals, cell lines, and procedures used in this study were reviewed and approved by the Ethics Committee of Tongji Hospital, Tongji Medical College, Huazhong University of Science and Technology before the initiation of the study. All animal experiments were conducted in compliance with the Animal Research: Reporting of In Vivo Experiments (ARRIVE) guidelines. Ethical approval for the use of human samples was obtained from the Ethics Committee of Tongji Hospital, Tongji Medical College, Huazhong University of Science and Technology (approval no. TJH IRB 20230839).

## Author Contributions

H.Z., Y.H., and J.W. conceived and supervised the project. Y.L. and Y.H. isolated and characterized primary cells. X.Z., Y.N., and Y.L. performed the in vivo mouse experiments. H.P. prepared and characterized the nanoparticles. Y.L., X.Z., and H.K. performed the in vitro experiments. J.C., L.H., T.L., and C.G. processed the data. Y.H. and X.Z. performed statistical analyses and drafted the manuscript. J.L. contributed to the interpretation and presentation of the results and critically revised the manuscript for important intellectual content. H.Z. and Y.H. had access to group allocation, whereas the remaining experimenters were blinded until data analysis was completed. All authors read and approved the final manuscript.

## Funding

The present study was supported by the National Natural Science Foundation of China (grant no. 82371405) and the Interdisciplinary Research Support Program of Huazhong University of Science and Technology (grant no. 5003540170).

## Conflicts of Interest

The authors declare no conflicts of interest.

## Supporting information




**Supporting File 1**: advs77972‐sup‐0001‐SuppMat.docx.

**Supporting File 2**: advs77972‐sup‐0002‐SuppMat.zip.

**Supporting File 3**: advs77972‐sup‐0003‐SuppMat.xlsx.

**Supporting File 4**: advs77972‐sup‐0004‐SuppMat.xlsx.

**Supporting File 5**: advs77972‐sup‐0005‐SuppMat.xlsx.

## Data Availability

All data needed to evaluate the conclusions in the paper are present in the paper and/or the Supplementary Materials. The newly generated single‐cell RNA‐seq data have been deposited in GSA under accession subCRA065396. Public datasets reanalyzed in this study are available from GEO under accessions GSE175430, GSE209552, GSE125976, GSE271769, GSE226207, GSE266610, and GSE180862. Materials are available from the corresponding author pending reasonable request and completion of any required material transfer agreement.
