# Supplementary material for: AI‐Designed TREM1‐Targeted LYTAC Nanoparticles Reprogram the Neuroimmune Microenvironment in Traumatic Brain Injury
Source: Adv Sci (Weinh). 2026 Sep 27:e77972. Online ahead of print. doi: 10.1002/advs.77972 (PMC13616302; doi:10.1002/advs.77972)
Supplement: Supplementary file 1 [file ADVS-9999-e77972-s004.docx]

**Supplementary Figures**

**Figure S1. Flow Cytometry Gating strategy**

a, Representative flow cytometry plots showing the gating strategy and TREM1 expression on CD45+CD11b+ myeloid cells in the peripheral blood of TBI patients.

b, Gating strategy for identifying TREM1+ cells within the CD45+CD11b+ microglia/macrophage population in mouse brain single-cell suspensions after excluding dead cells.

c, Flow cytometric analysis of TREM1 expression on CD45+CD11b+ cells in mouse peripheral blood.

d, Ly6C expression within the TREM1+ cell subset in mouse brain tissue.

e, CCR2 (chemokine receptor) expression on TREM1+ cells in mouse brain tissue.

f, Analysis of TREM1 and CCR2 co-expression within the CD45+CD11b+ population in brain tissue samples from TBI patients.

**Figure S2. Generation of Trem1 knockout mice and additional analyses of neuroinflammatory injury**

a, Gene typing results of homozygous, heterozygous and wild-type mice according to Shanghai Model Organisms Center’s instruction.

b, Quantitation of focused inflammatory cytokines detected by The Proteome Profiler Array Mouse Cytokine Array Panel A in WT or *Trem1*^-/-^ mice after TBI 3d in Figure 2b.

c, Representative flow cytometry plots and quantification of infiltrating CD45^hi^CD11b^+^ myeloid cells in injured brain tissues from WT and *Trem1*^−/−^ mice at 3 d after TBI.

d, Representative images of Iba1 and TNFa and quantitation in mice cortex 3 d post-TBI (n = 4 mice per group; unpaired Student’s t test, WT TBI vs. *Trem1*^−/−^ TBI, ****P* = 0.0001, scale bar, 50 µm).

e, Quantitation of CD86 MFI in mice cortex 3 d post-TBI in Figure 2d (n = 5 mice per group; unpaired Student’s t test, WT TBI vs. *Trem1*^−/−^ TBI, ****P* = 0.0001).

f, Quantification of the percentage of TUNEL-positive cells colocalized with NeuN (n = 6 mice per group, unpaired Student’s t-test, ****P* = 0.0006).

g, Quantitation of MBP Mean Fluorescence Intensity (MFI) in mice cortex 3 d post-TBI in Figure 2f (n = 6 mice per group; unpaired Student’s t test, WT TBI vs. *Trem1*^−/−^ TBI, ****P* = 0.0003).

h, Quantitation of Neun^+^ cell counts in mice hippocampus 3 d post-TBI in Figure 2g.

**Figure S3. Flow Cytometry validation of bone marrow transplantation**

a, FC analysis (left panel) and (right panel) quantitation of CD45^+^CD11b^+^TREM1^+^ cells in BM in TBI mice after bone marrow transplantation or sham (n = 3 mice per group, one-way ANOVA, *Trem1*^-/-^ TBI vs. WT BMT-*Trem1*^-/-^ TBI, *****P* < 0.0001; WT BMT-WT TBI vs. WT BMT-*Trem1*^-/-^ TBI, ***P* = 0.0098).

b, FC analysis (left panel) and (right panel) quantitation of CD45^+^CD11b^+^TREM1^+^ cells in Blood in TBI mice after bone marrow transplantation or sham (n = 3 mice per group, one-way ANOVA, *Trem1*^-/-^ TBI vs. WT BMT-*Trem1*^-/-^ TBI, *****P* < 0.0001; WT BMT-WT TBI vs. WT BMT-*Trem1*^-/-^ TBI, ***P* = 0.0026).

c, FC analysis (left panel) and (right panel) quantitation of CD45^+^CD11b^+^TREM1^+^ cells in Brain in TBI mice after bone marrow transplantation or sham (n = 3 mice per group, one-way ANOVA, *Trem1*^-/-^ TBI vs. WT BMT-*Trem1*^-/-^ TBI, *****P* < 0.0001; WT BMT-WT TBI vs. WT BMT-*Trem1*^-/-^ TBI, **P* = 0.0171).

**Figure S4. AlphaFold-based structural prediction of Pep457 binding to human TREM1.**

a, Predicted structural model of the Pep457–human TREM1 ectodomain complex. Human TREM1 is shown in pink and Pep457 in purple. The enlarged view highlights the predicted binding interface and selected intermolecular polar contacts between Pep457 and human TREM1; dashed lines indicate predicted hydrogen-bonding or polar interactions. This computational model supports the structural feasibility of Pep457 engagement with human TREM1, although direct experimental validation of human TREM1 binding will be required.

**Figure S5. M6P conjugation preserves the TREM1-binding activity of Pep457.**

a, Concentration-dependent binding curves of the unconjugated TREM1 binder (left) and the M6P-conjugated TREM1 binder (TREM1-binder-M6P₃; right) to immobilized TREM1. Binding was quantified as fraction bound and fitted using nonlinear regression. The comparable binding profiles indicate that M6P conjugation did not substantially impair the TREM1-binding activity of the Pep457 binder. Data are presented as mean ± SD.

**Figure S6. ^1^H NMR characterization and serum stability of APTL-NP.**
a, Representative ^1^H NMR spectra of G4 PAMAM and APTL-NP. Characteristic resonance regions are annotated for PAMAM methylene protons (δ 2.4–3.0 and 3.1–3.4 ppm), aromatic protons derived from Angiopep-2, Pep457, and TPA (δ 6.8–7.3 ppm), and overlapping resonances attributable to Pep457 α-CH, M6P, Angiopep-2, and PAMAM protons (δ 3.20–3.98 ppm). The residual HDO signal is indicated at δ 4.79 ppm. These spectral features support successful multicomponent functionalization of the PAMAM-based nanoplatform.
b, Time-dependent hydrodynamic diameter and polydispersity index (PDI) of APTL-NP during incubation in 50% fetal bovine serum (FBS) at pH 7.4 and 37 °C for up to 72 h, demonstrating maintenance of colloidal stability under serum-containing conditions. Data are presented as mean ± SD (n = 3 independent measurements).

**Figure S7. Temporal acidification of the TBI lesion microenvironment.**

a, Schematic of intralesional pH measurement after traumatic brain injury.
b, Lesion pH measured in sham-operated mice and at the indicated time points after TBI. Each dot represents one animal.

**Figure S8. Expanded assessment of systemic compatibility after APTL-NP administration.**

a,b, Plasma C5a and C3a concentrations in normal mice and in sham- or TBI-operated mice treated with vehicle or APTL-NP at the indicated time points.
c, Complete blood count analysis of circulating white blood cells, neutrophils, monocytes, and lymphocytes in sham- and TBI-operated mice treated with vehicle or APTL-NP.
d–j, Serum biochemical indices measured at 24 h and 7 d after treatment, including creatinine (CREA), alanine aminotransferase (ALT), aspartate aminotransferase (AST), alkaline phosphatase (ALP), total bilirubin (TBIL), albumin (ALB), and blood urea nitrogen (BUN).

Data are presented as mean ± SD. Individual points represent independent biological replicates.

**Figure S9. Evaluation of endothelial barrier integrity after nanoparticle treatment in the in vitro BBB model.**

a, TEER values of the bEnd.3 endothelial monolayer measured over 24 h after treatment with Binder, TREM1-LYTAC, NP-TREM1-LYTAC, APTL-NP, or Angiopep-2-preblocked APTL-NP. TEER values remained stable among groups, indicating that APTL-NP-mediated trans-BBB delivery was not caused by overt disruption of endothelial barrier integrity. Data are presented as mean ± SD, n = 3; two-way ANOVA.

**Figure S10. Quantitative analysis of in vivo and ex vivo biodistribution corresponding to Figure 5i and j.**
a**,** Quantification of head-region Cy5.5 fluorescence in TBI mice receiving Cy5.5-labeled Binder or APTL-NP at 12 h, 1 d, and 3 d after administration (n = 6 mice per group). Fluorescence intensity is expressed as total flux (photons s⁻¹). b**,** Quantification of ex vivo Cy5.5 fluorescence in the brain, heart, lung, liver, spleen, kidney, and intestine at the indicated time points (n = 3 mice per time point). Fluorescence intensity is expressed as total flux (photons s⁻¹). Data are presented as mean ± SD, with each dot representing an individual animal. Statistical significance was assessed by two-way ANOVA followed by Sidak’s multiple-comparison test. **P < 0.01, ***P < 0.001, ****P < 0.0001; comparisons are indicated by brackets.

**Figure S11. Comparison of therapeutic outcomes among sham, TBI vehicle-treated, and APTL-NP-treated mice.**
a**,** Representative images of Evans blue extravasation in brains from Sham + Vehicle, TBI + Vehicle, and TBI + APTL-NP groups. b**,** Quantification of Evans blue extravasation in the indicated groups (n = 6 mice per group). c**,** Brain water content in the indicated groups as an assessment of cerebral edema (n = 6 mice per group). d**,** Levels of the indicated inflammation-associated cytokines and mediators in brain tissue from Sham + Vehicle, TBI + Vehicle, and TBI + APTL-NP groups. Data are from three independent experimental replicates. e**,** Motor performance assessed using the fixed-speed rotarod test at 30 rpm, expressed as latency to fall (n = 6 mice per group). f**,** Motor performance assessed using the accelerating rotarod test (4–40 rpm over 300 s) at the indicated time points after TBI, expressed as latency to fall (n = 6 mice per group, * indicate comparisons between Sham + Vehicle and TBI + APTL-NP, # indicate comparisons between TBI + Vehicle and TBI + APTL-NP).

Data are presented as mean ± SD. Each dot in b, c, and e represents an individual animal, whereas each dot in d represents an independent experimental replicate. Statistical significance in b–e was assessed by one-way ANOVA followed by Tukey’s multiple-comparison test, and that in f by two-way ANOVA followed by Tukey’s multiple-comparison test. *P < 0.05, **P < 0.01, and ****P < 0.0001; ##P < 0.01 and ###P < 0.001, with specific comparisons indicated in the figure.

**Figure S12. APTL-NP does not confer additional neurovascular or functional benefit in Trem1-deficient mice after TBI.**

a, Representative images of Evans blue extravasation in the injured brains of Trem1-deficient mice treated with Vehicle, G4, LP17, scrambled-LYTAC, free TREM1-LYTAC, or APTL-NP after TBI.

b, Quantification of Evans blue extravasation in the indicated treatment groups.

c, Brain water content in Trem1-deficient mice after the indicated treatments.

d, Performance in the accelerating rotarod test at days 1, 3, and 7 after TBI.

e, mNSS assessed at days 1, 3, and 7 after TBI.

Data are presented as mean ± SD. Individual points represent individual animals. Statistical analyses are described in the Methods.

**Figure S13. Identification and characterization of major cell types and subtypes.**

a, Dot plot showing the expression of canonical marker genes used to annotate the major cell clusters.

b–d, Dot plots displaying the expression of distinct signature genes used to define subtypes within specific clusters: (b) Microglia and Macrophage subtypes, categorizing clusters into Homeostatic, Disease-associated microglia (DAM)/Lipid-metabolism, Pro-inflammatory (M1-like), Anti-inflammatory (M2-like), and Proliferating states. (c) Astrocyte subtypes, distinguishing between Homeostatic, Pan-reactive, Pro-inflammatory (A1-like), Neuroprotective (A2-like), and Scar-forming phenotypes. (d) Endothelial and stromal cell subtypes, highlighting Blood-Brain Barrier (BBB) integrity, Inflammatory activation, and Pericyte/Fibroblast subpopulations.

**Figure S14. APTL-NP reshapes intercellular communication networks in TBI cortex.**

a, CellChat analysis visualizing the global intercellular communication network. The chord diagrams illustrate the aggregate strength of ligand-receptor interactions, showing a reorganized connectivity pattern in APTL-NP brains compared to Vehicle.

b,Heatmaps showing the differential number of interactions (left) and differential interaction strength (right) between the APTL-NP and Vehicle groups.

c,Stacked bar plots summarizing the information flow of significant signaling pathways. The left panel shows the relative information flow (normalized contribution of each group), and the right panel shows the total information flow (overall signaling strength) for each pathway.

**Supplementary Table 1: Patient clinical parameters**

|  | Age | Gender (M1, F0) | Type | GCS | MMSE | Monocyte  Count | Neutrophil  Count | sTREM1 |
| --- | --- | --- | --- | --- | --- | --- | --- | --- |
| 1 | 49 | 1 | Severe | 7 | 0 | 0.94 | / | 864.23 |
| 2 | 39 | 1 | Severe | 8 | 3 | 1.08 | 9.03 | 758.43 |
| 3 | 72 | 1 | Severe | 6 | 0 | 0.56 | 10.08 | 722.77 |
| 4 | 62 | 0 | Severe | 8 | 10 | 0.92 | 12.1 | 703.75 |
| 5 | 34 | 1 | Moderate | 10 | 15 | 0.97 | 6.94 | 667.61 |
| 6 | 53 | 1 | Moderate | 9 | 9 | 0.79 | 11.04 | 654.67 |
| 7 | 57 | 1 | Moderate | 9 | 11 | 1 | 10.82 | 583.79 |
| 8 | 43 | 0 | Severe | 8 | 13 | 0.81 | 8.3 | 574.92 |
| 9 | 62 | 1 | Severe | 6 | 0 | 0.75 | 12.8 | 572.59 |
| 10 | 45 | 1 | Severe | 8 | 17 | 1.52 | 10.8 | 566.75 |
| 11 | 60 | 1 | Severe | 8 | 11 | 0.76 | 9.4 | 485.13 |
| 12 | 57 | 0 | Moderate | 10 | 23 | 0.72 | 12.53 | 425.68 |
| 13 | 53 | 0 | Moderate | 9 | 13 | 0.68 | 6.84 | 404.15 |
| 14 | 57 | 1 | Moderate | 10 | 17 | 1.17 | 8.11 | 389.31 |
| 15 | 48 | 0 | Moderate | 11 | 19 | 0.67 | 9.94 | 373.07 |
| 16 | 39 | 1 | Severe | 7 | 20 | 0.75 | 6.84 | 369.14 |
| 17 | 46 | 1 | Severe | 6 | 4 | 0.86 | 13.81 | 302.84 |
| 18 | 54 | 1 | Moderate | 9 | 16 | 0.7 | 10.44 | 269.43 |
| 19 | 44 | 0 | Moderate | 12 | 8 | 0.71 | 6.72 | 268.13 |
| 20 | 32 | 1 | Moderate | 12 | 13 | 0.65 | 8.1 | 259.54 |
| 21 | 29 | 1 | Severe | 8 | 9 | 0.82 | 10.7 | 248.71 |
| 22 | 41 | 0 | Moderate | 10 | 25 | 0.72 | 6.84 | 246.09 |
| 23 | 57 | 1 | Moderate | 11 | 21 | 0.63 | 8.81 | 207.03 |
| 24 | 37 | 1 | Moderate | 12 | 20 | 0.83 | 8.98 | 179.36 |
| 25 | 58 | 1 | Severe | 7 | 0 | 0.68 | 7.6 | 178.75 |
| 26 | 47 | 0 | Severe | 6 | 18 | 0.51 | 9.7 | 175.27 |
| 27 | 59 | 0 | Moderate | 9 | 5 | 0.64 | 8.8 | 171.38 |
| 28 | 37 | 1 | Moderate | 12 | 18 | 0.49 | 9.11 | 149.43 |
| 29 | 36 | 1 | Moderate | 9 | 7 | 0.42 | 7.25 | 148.90 |
| 30 | 42 | 1 | Moderate | 11 | 17 | 0.61 | 8.34 | 62.91 |
| 31 | 28 | 1 | / |  |  |  |  |  |
| 32 | 43 | 0 | / |  |  |  |  |  |
| 33 | 56 | 0 | / |  |  |  |  |  |
| 34 | 49 | 1 | / |  |  |  |  |  |
| 35 | 57 | 1 | / |  |  |  |  |  |
| 36 | 63 | 0 | / |  |  |  |  |  |
| 37 | 55 | 0 | / |  |  |  |  |  |
| 38 | 25 | 1 | / |  |  |  |  |  |
| 39 | 34 | 1 | / |  |  |  |  |  |
| 40 | 52 | 1 | / |  |  |  |  |  |

**Supplementary Information 1**

Synthesis of APTL-NP

*Materials*

Generation 4 (G4) PAMAM dendrimers, terephthalaldehyde (TPA), 1-ethyl-3-(3-dimethylaminopropyl) carbodiimide (EDC), N-hydroxysuccinimide (NHS), sodium cyanoborohydride (NaBH3CN), and PS-PEG-NHS were purchased from Aladdin (Shanghai, China). The TREM-1 specific antagonist peptide and Angiopep-2 were custom-synthesized by Wuhan Mabnus Biotechnology Co., Ltd. (Wuhan, China). All general reagents, including dimethyl sulfoxide (DMSO), phosphate-buffered saline (PBS), and 2-(N-morpholino) ethanesulfonic acid (MES), were of analytical grade and used as received. Purification was performed using dialysis membranes (MWCO 3.5 kDa) obtained from Thermo Scientific™.

*Synthesis of PAMAM-TPA Conjugates*

G4 PAMAM was functionalized with terephthalaldehyde (TPA) to introduce aldehyde-bearing linkers for subsequent acid-labile payload conjugation. Briefly, 20 mg of G4 PAMAM was dissolved in 1 mL of PBS (pH 7.2). Separately, 30.2 mg of TPA was dissolved in 140 μL of DMSO by ultrasonication for 10 s. A G4 PAMAM/DMSO solution was prepared by adding 200 μL of the G4 PAMAM aqueous solution to 5.8 mL of DMSO. Under dark conditions at room temperature, 20 μL of the TPA/DMSO solution was added dropwise to 3 mL of the G4 PAMAM/DMSO solution over 30 min, followed by gentle stirring for 2 h.

To generate a stable PAMAM-TPA intermediate while retaining free aldehyde groups for payload conjugation, the Schiff base formed between PAMAM surface amines and one aldehyde group of TPA was selectively stabilized by reductive amination using NaBH₃CN. The reaction mixture was dialyzed against deionized water at 4 °C for 24 h using a dialysis membrane with a molecular weight cutoff of 3.5 kDa, with water changed every 4 h to remove unreacted TPA and small molecules. The purified PAMAM-TPA product was obtained by lyophilization.

*Conjugation of TREM1-LYTAC*

TREM1-LYTAC was conjugated to PAMAM-TPA through the remaining aldehyde groups of TPA to generate an acid-labile Schiff base linkage. Briefly, lyophilized PAMAM-TPA 3 mg and TREM1-LYTAC 5 mg were dissolved in 5 mL of PBS pH 7.2. The mixture was stirred gently at room temperature for 4–6 h to allow Schiff base formation between amine groups on TREM1-LYTAC and residual aldehyde groups on the TPA linker. Importantly, this TPA–TREM1-LYTAC imine linkage was not reduced, thereby preserving its acid-labile property for pH-responsive payload release under acidic conditions.

After the reaction, the solution was dialyzed against deionized water at 4 °C for 24 h using a dialysis membrane with a molecular weight cutoff of 3.5 kDa to remove unconjugated TREM1-LYTAC. The purified product was designated PAMAM-TPA-TREM1-LYTAC.

*Conjugation of* *Angiopep-2*

Angiopep-2 was covalently attached to residual PAMAM surface amines through EDC/NHS-mediated coupling. Briefly, 10 mg of Angiopep-2 peptide was dissolved in 5 mL of MES buffer pH 5.5–6.0. Carboxyl groups on Angiopep-2 were activated by adding 20 mg of EDC and 20 mg of NHS, followed by pre-activation for 30 min. An additional 10 mg of EDC was supplemented every 10 min to maintain activation efficiency. Subsequently, 3 mg of PAMAM-TPA-TREM1-LYTAC was dissolved in 5 mL of PBS pH 7.2–7.8. The activated Angiopep-2 solution was slowly added to the PAMAM-TPA-TREM1-LYTAC solution in three aliquots, and the reaction was stirred at room temperature for 4–12 h or overnight. The resulting mixture was dialyzed against deionized water at 4 °C for 24 h using a dialysis membrane with a molecular weight cutoff of 3.5 kDa to remove byproducts and unreacted peptides. The purified product was designated Angiopep-2/PAMAM/TPA/LYTAC nanoparticle, or APTL-NP.

*Surface PEGylation and Final Assembly*

To enhance water solubility, prolong blood circulation time, and introduce the targeting moiety, the nanoparticle surface was modified with PS-PEG-NHS. The PAMAM-TPA-TREM1-LYTAC- Angiopep-2 conjugate (10 mg) and PS-PEG-NHS (35 mg, adjusted based on the desired degree of PEGylation) were dissolved in 5 mL of PBS. The reaction was performed in a round-bottom flask with gentle stirring at room temperature for 3–6 h (or overnight). The NHS-ester of the PEG derivative reacted selectively with the remaining primary amines on the PAMAM surface to form stable amide bonds. The final product was purified by dialysis (MWCO 3.5 kDa) at 4°C for 24 h against deionized water to remove free PEG and other impurities. The final construct, PAMAM-TPA-TREM1-LYTAC-Ang2-PEG (APTL-NP), was lyophilized and stored at -20°C.

Physicochemical Characterization

1) Solubility Profile:

Exhibits excellent aqueous solubility: 10 mg/mL, test conditions: room temperature (25°C) in PBS buffer (pH 7.2); and maintains stable dissolution characteristics across multiple batches;

2) Morphology & Storage Stability:

Lyophilized form: white, free-flowing powder

Storage conditions: Long-term: -20 °C; Short-term: 4 °C in aqueous solution (stable for ≥1 month)

Reconstitution properties: rapid dissolution in aqueous media (<30 seconds with gentle vortexing)

3) Characterization and analysis results of nanoparticles:

(1) Hydrogen nuclear magnetic resonance (¹H NMR) spectroscopy characterization revealed distinct spectral patterns for both the PAMAM dendrimer carrier and the final APTL-NP conjugate. The fourth-generation G4 PAMAM dendrimer exhibited characteristic proton resonances predominantly in the 2.5-3.0 ppm range, with minor peaks appearing between 3.1-3.5 ppm. In contrast, the APTL-NP conjugate showed a broader chemical shift range (2.5-5.0 ppm) while maintaining the characteristic PAMAM peaks, with additional resonances corresponding to conjugated components.

(2) Transmission electron microscopy (TEM) analysis showed that the PAMAM-based precursor particles had an average TEM-measured diameter of 159 ± 34 nm, whereas APTL-NP showed a slightly larger diameter of 191 ± 24 nm.

(3) Dynamic light scattering (DLS) analysis revealed distinct size distributions for the nanoparticle formulations. The PAMAM dendrimer exhibited a hydrodynamic diameter of 153 ± 22 nm, while the APTL-NP conjugate demonstrated a slightly larger diameter of 187 ± 17 nm.
